# Resilience-related neural similarity during naturalistic movie watching

**DOI:** 10.1162/IMAG.a.1165

**Published:** 2026-03-16

**Authors:** Shuer Ye, Leona Rahel Bätz, Avneesh Jain, Alireza Salami, Maryam Ziaei

**Affiliations:** Kavli Institute for Systems Neuroscience, Norwegian University of Science and Technology, Trondheim, Norway; Umeå Center for Functional Brain Imaging (UFBI), Umeå University, Umeå, Sweden; Aging Research Center, Karolinska Institutet & Stockholm University, Stockholm, Sweden; Department of Medical and Translational Biology, Umeå University, Umeå, Sweden; Department of Psychology, Florida State University, Tallahassee, FL, United States

**Keywords:** movie-fMRI, resilience, inter-subject correlation, intolerance of uncertainty, neural similarity

## Abstract

Psychological resilience protects individuals against the negative consequences of exposure to adversity. Despite increasing attention given to resilience for its role in maintaining mental health, a clear conceptualization of resilience remains elusive, and the intricacies of its neural correlates are poorly understood. Here, we recorded brain activity in healthy young adults using a 7T MRI scanner while they naturally watched two movie clips, one with neutral content and another with negative content. Stronger and more extensive neural similarity, as estimated by inter-subject correlation, was observed in response to the negative movie compared with the neutral movie. Moreover, we found that high-resilience individuals had similar neural activities to their peers, while low-resilience individuals showed more variable neural activities. A secondary analysis examined the relationship between ISCs and an additional self-report behavioral measure of interest, Intolerance of Uncertainty (IU). IU is a personality trait known to bias perception and cognition and has been proposed to be related to resilience. We found that higher IU was associated with attenuated resilience-related neural similarity in attention-related brain regions, suggesting that IU may undermine resilience by altering attentional focus toward specific aspects of the external environment. We propose that the similarity of neural responses among resilient individuals highlights adaptive emotional processing engaging multiple brain systems. Conversely, the variability in neural responses among low-resilience groups indicates vulnerability to adverse psychological outcomes. These insights shed light on the mechanisms of resilience, highlighting that it involves a constellation of neuropsychological processes crucial for adapting to external stimuli.

## 1 Introduction

Psychological resilience, the ability to cope effectively with adversities, plays a crucial role in determining individuals’ overall well-being (Feldman, 2020). Although resilience was conceptualized as a stable trait for decades, researchers increasingly recognize it as a dynamic process encompassing positive adaptation in the face of negative events (Denckla et al., 2020; Rutter, 2012; Vella & Pai, 2019). The key role of resilience lies in altering stress responses, achieved through the accurate perception of stressors, evaluation of both situational demands and coping resources, and the mobilization of adaptive strategies, ultimately mitigating the adverse impact of stress and resisting the development of illness or psychological distress (Dedovic et al., 2009; Parsons et al., 2016; Schäfer, 2015; Schetter & Dolbier, 2011). Individuals with higher resilience tend to report more positive emotional experiences (Tugade et al., 2004), demonstrate quicker disengagement from emotional stimuli (Yi et al., 2020), and employ adaptive emotional regulation strategies more effectively (Polizzi & Lynn, 2021). Neuroimaging evidence has demonstrated that resilience is largely supported by the brain’s emotion-related systems that are involved in critical emotional and cognitive processing such as attentional modulation (Jääskeläinen et al., 2021), emotion perception (Homberg & Jagiellowicz, 2022), emotion regulation (Liu et al., 2023), and executive control (Rodman et al., 2019). Sustaining the functioning of these systems is essential for supporting resilience and enabling adaptive responses following exposure to stressors (Brunetti et al., 2017; Roeckner et al., 2021).

Previous studies concerning the neural correlates of resilience have predominantly relied on resting-state paradigms (Kraft & Fiebach, 2022; Liu et al., 2023) or highly controlled tasks (Long et al., 2019; Miyagi et al., 2020; Shi et al., 2019). While these approaches offer valuable insights into underlying neural mechanisms, their replicability and ecological validity are limited (Finn, 2021). In contrast to traditional paradigms, naturalistic designs such as movie-fMRI capture complexity and dynamics that are relatively close to real-world experiences (Hamilton & Huth, 2020; Lenormand & Piolino, 2022; Meer et al., 2020). This approach not only enhances ecological validity but also accentuates interindividual variability, offering a powerful framework to examine how the brain processes complex stimuli while overcoming the constraints of resting-state design and highly controlled experimental tasks (Nastase et al., 2020; Sonkusare et al., 2019; Vanderwal et al., 2017). By recording brain activity while participants watched movies, inter-subject correlation (ISC) was computed as the correlation of the BOLD-signal time courses within corresponding brain regions across participants (Gerardin et al., 2025; Noad et al., 2025; Nummenmaa et al., 2018; Salmi, 2020). Higher ISC values indicate greater temporal synchronization of neural responses in a given region, reflecting increased neural similarity and suggesting that individuals may process information in a similar manner (Hasson et al., 2010). Notably, emotionally charged content tends to elicit greater neural similarity (Hasson, 2008). Heightened neural similarity may stem from increased attentional engagement induced by the content of the movie (Ki et al., 2016) and the recruitment of specialized brain circuits involved in processing salient and emotional contents (Cooper et al., 2021). Consistent with this view, empirical evidence shows that negative clips reliably increase arousal, inducing negative affect, and activate stress-related responses, such as increased salivary cortisol levels, reflecting strong autonomic and HPA-axis activation (Cousijn et al., 2012; Everaerd et al., 2015; Henckens et al., 2016; van Marle et al., 2010).

While behavioral studies have suggested that resilient individuals may share similar patterns of perceiving and processing external stimuli (Polizzi & Lynn, 2021; Tugade et al., 2004), no study to date has examined whether these behavioral similarities among resilient individuals are also expressed at the neural level during naturalistic processing. Recent movie-fMRI studies in clinical populations have demonstrated reduced similarity in neural responses among patients with attention deficits and hyperactivity disorder (Salmi, 2020), schizophrenia (Tu, 2019), and depression (Gruskin et al., 2020). In contrast, socially well-connected individuals have been shown to exhibit more similar brain responses during movie viewing (Baek et al., 2022). Likewise, more optimistic individuals display shared neural representations when engaging in episodic future thinking (Yanagisawa et al., 2025). These findings suggest that convergent neural activity may be associated with positive psychological traits (Kleiman et al., 2017; Nitschke et al., 2021). Conversely, divergent or less similar neural responses have been associated with vulnerability to negative psychological outcomes (Wei et al., 2025; Zhang et al., 2024). This pattern is in line with the Anna Karenina (AnnaK) model (Finn, 2020; Ohad & Yeshurun, 2023), inspired by the opening line of Leo Tolstoy’s Anna Karenina novel, which posits that “all happy families are alike; but each unhappy family is unhappy in its own way” (Tolstoy, 1997). In the context of the present study, we aim to examine whether individuals with high resilience scores exhibit greater similarity in their neural responses to naturalistic movie stimuli, whereas those with low resilience scores show more heterogeneous or idiosyncratic response patterns.

Moreover, to explore psychological mechanisms that might underlie resilience-related neural similarity, we conducted a secondary analysis examining intolerance of uncertainty (IU) as a candidate factor (Tanovic et al., 2018). IU is a personality trait indicating a tendency to avoid uncertainty and harbor negative beliefs about ambiguous or uncertain situations (Carleton, 2012; Grenier et al., 2005) and plays a role in gating attentional engagement (Dieterich et al., 2016; Sahib et al., 2023) and shaping emotion perception (Perlman, 2009). IU is also a risk factor for generalized anxiety disorder (Gu et al., 2020). By incorporating IU, we were able to test whether disruptions in attentional regulation and emotion perception were linked to reduced resilience-related neural similarity, thereby providing deeper insight into the underlying neuropsychological mechanisms of resilience.

Thus, this study aimed to investigate resilience-related neural similarity during naturalistic processing by using movie-fMRI. Our primary objectives were twofold: first, to scrutinize the impact of resilience on individuals’ neural responses by employing naturalistic stimuli and intersubject representational similarity analysis (IS-RSA, G. Chen et al., 2017), a novel ISC-based approach to robustly detect individual differences and the underlying brain dynamics in naturalistic settings. Second, to identify the association between IU, resilience and neural similarity during movie watching. To achieve these goals, we utilized fMRI data from a cohort of young healthy adults with varying levels of self-report resilience scores. Participants were scanned while watching two types of movie clips: a neutral clip with low emotional content and a negative clip that has been shown to elicit strong emotional and stress responses. Based on the literature (Baek et al., 2022; Finn, 2020), we hypothesized that individuals with high resilience display more consistent neural responses, while those with low resilience exhibit more idiosyncratic neural responses, which would support the AnnaK model. We also expected this effect to be more pronounced in the negative movie condition, as negative emotional content may evoke stronger emotional reactions and stress responses that engage resilience-related processes. Moreover, we anticipated that IU, as a vulnerability factor contributing to reduced resilience, would attenuate the resilience-related neural similarity, especially in the brain areas responsible for attention and emotion perception.

## 2 Materials and Methods

### 2.1 Participants

Seventy-eight younger adults were recruited from the local community via paper flyers and online advertisements to participate in this study. All participants were healthy, right-handed, and reported no history of neurological (e.g., epilepsy, stroke, or brain injury) or psychiatric disorders (e.g., depression, anxiety, schizophrenia, or autism spectrum disorder) within the past 5 years. Participants were screened for current use of psychotropic or neurological medications, and no participants met the exclusion criteria or were excluded on this basis at the time of the experiment. Due to technical issues, incomplete data, and excessive movement (i.e., average framewise displacement [FD] > 0.25 mm), 62 participants (33 males and 29 females, age range 19–35 years, mean age 25.68 ± 4.30 years) were included for further data analyses. All participants provided written informed consent and were compensated with a gift card for their participation. The study was approved by the Regional Committee for Medical Research Ethics in Central Norway (REK Midt, approval #390390).

### 2.2 Procedure

**Fig. 1.**
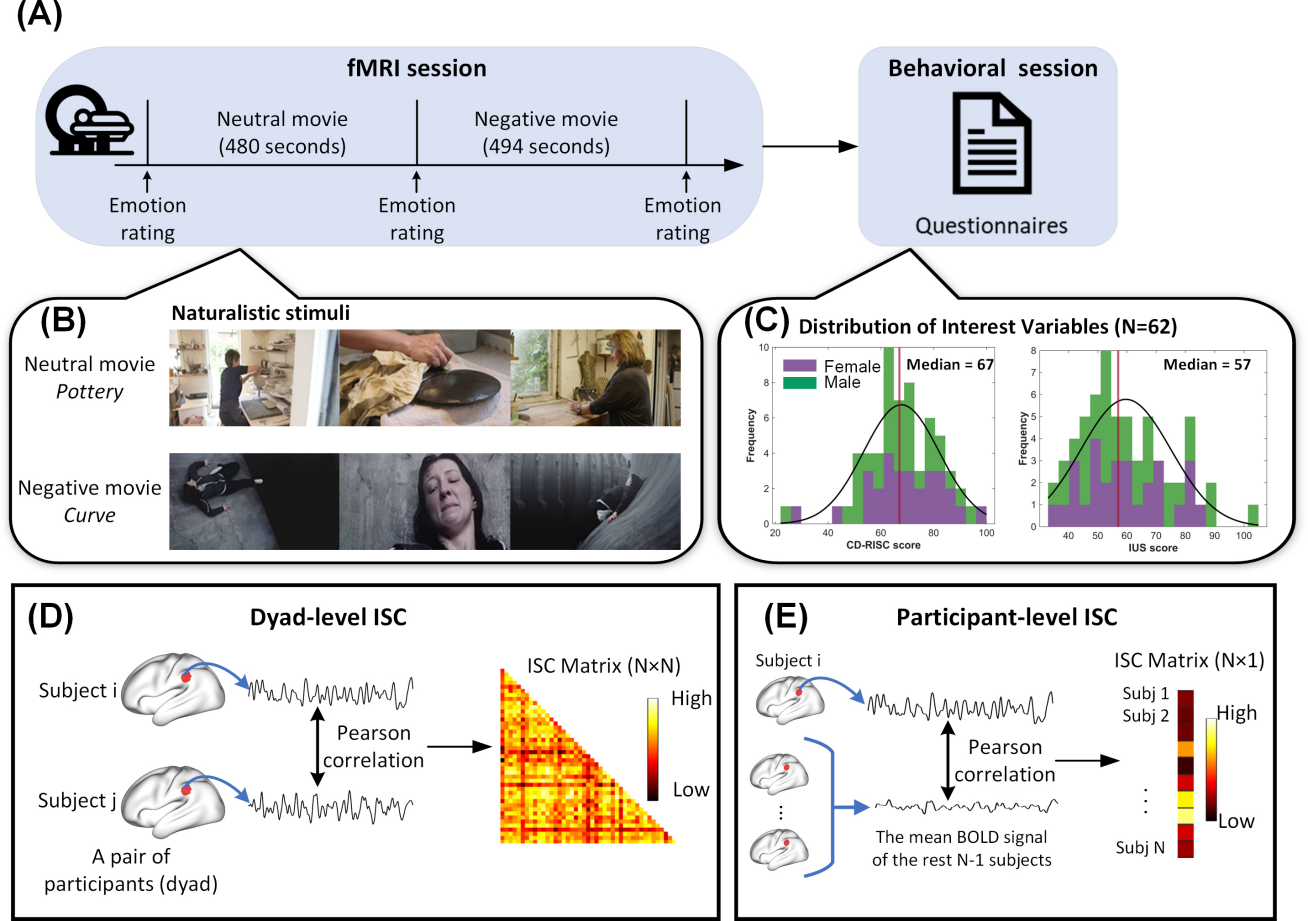
Study paradigm and calculation of inter-subject correlation (ISC). (A) Participants first underwent the fMRI scanning while they watched two movies. Before and after each movie, they reported their emotional valence and arousal. After the fMRI session, participants were invited back to complete a behavioral session comprising self-reported questionnaires and cognitive testing as part of the protocol for a larger study on resilience and aging brain. (B) Participants first watched the neutral movie, “*Pottery*”, and then the negative movie, “*Curve*”, in the 7T MRI scanner. (C) Histogram plots for resilience score measured by Connor–Davidson Resilience Scale (CD-RISC) and intolerance to uncertainty score measured by Intolerance of Uncertainty Scale (IUS). Scores of both questionnaires are normally distributed. (D) Dyad level ISC was computed by correlating the BOLD signal time series of the two participants within each dyad. (E) Participant-level ISC was computed by correlating the BOLD signal time series of the given participant and the mean BOLD signal time series of the rest of participants. The inferior parietal lobe is depicted here as an example for the analyses.

The study consisted of two sessions: a brain imaging session and a behavioral session (Fig. 1A). First, participants took part in an MRI session, which included both functional and structural brain imaging protocols. Following the brain imaging session, the behavioral session took place within 2 days and lasted about 2 hours, involving self-reported questionnaires and computerized tasks.

Before the brain imaging session, participants received instructions about the procedure and the experiment, both verbally and in written format. During the scan, participants underwent an 8-minute anatomical scan followed by a movie-watching task. They were presented with a neutral movie, followed by a negative movie, and were asked to rate their emotional valence and arousal levels before and after each movie clip by verbally reporting their responses on a 9-point Self-Assessment Manikin scale. Employing fixed movie sequences ensured consistent stimulus context across participants and effectively eliminated confounding factors arising from variability in stimulus presentation (Baek et al., 2022). Participants were instructed to watch the videos naturally while trying to remain still during the scanning session. Foam padding was used to minimize head motion, and participants listened to the videos’ sound through earphones (BOLDfonic, Cambridge Research Systems Ltd) compatible with 7T MRI scanner while watching the videos through a mounted mirror on the head coil.

### 2.3 Movie clips

Prior to the main study, a pilot behavioral study was conducted to select materials for the main experiment. Four movies from prior studies and online repositories (Taylor et al., 2017) were chosen for the pilot experiment (Supplementary Table S1). Twenty-four young adults (12 males, mean age = 25.5 ± 3.96 years) provided a series of ratings of valence, arousal, and emotion types, as well as a continuous intensity rating, for each movie clip. Based on the results of the pilot study, two movie clips were chosen for the subsequent brain imaging study (more details of the pilot study are given in Supplementary Information, Fig. S1). The neutral movie, titled “*Pottery*”, depicts two women engaged in pottery making, which lasted for 480 seconds. The negative movie, named “*Curve*”, tells the story of a woman struggling to maintain her balance on the slope of a dam, desperately trying to avoid falling into the abyss below, which lasted for 494 seconds (Fig. 1B).

### 2.4 Questionnaires

As this study was part of a larger study, participants were asked to fill out a variety of self-reported questionnaires, including anxiety, depression, and perceived stress, and to complete computerized tasks, including theory of mind, Stroop, and emotion perception.

The level of psychological resilience was assessed using the Connor–Davidson Resilience Scale (CD-RISC), which is a well-validated scale to screen individuals for varying levels of resilience (Connor & Davidson, 2003) and has been used in a variety of settings including behavioral (van Gils et al., 2022), clinical (Hendricks et al., 2023), and neuroimaging studies (Long et al., 2019). The scale consists of 25 items such as “I am able to adapt when changes occur”. Each item is scored on a 5-point Likert scale from 0 (Not true) at all to 4 (True nearly all the time). Higher scores indicate a higher level of resilience. In the current study, the Cronbach’s Alpha of the CD-RISC is 0.902, reflecting high internal consistency and reliability in our sample.

The intolerance of uncertainty was assessed with the Intolerance of Uncertainty Scale (IUS) (Freeston et al., 1994), which comprises 27 items such as “Unforeseen events upset me greatly”, on a 5-point Likert scale from 1 (Not at all characteristic of me) to 5 (Entirely characteristic of me). Higher scores indicate increased intolerance of uncertainty. The Cronbach’s Alpha of the IUS is 0.904 in the current sample. Furthermore, we utilized the Hospital Anxiety and Depression Scale (Bjelland et al., 2002), Perceived Stress Scale (Lee, 2012), and Difficulties in Emotion Regulation Scale (DERS) (Gratz & Roemer, 2004) to evaluate participants’ levels of anxiety, depression, and stress, as well as their difficulties in emotion regulation.

### 2.5 Imaging data acquisition

The imaging data were collected on a 7T MRI Siemens MAGNETOM Terra scanner with a 32-channel head coil. Functional images were acquired using a multi-band accelerated echo-planar imaging sequence (92 interleaved slices, multi-band acceleration factor = 2, echo time = 19 ms, repetition time = 2000 ms, matrix size = 160 × 160 mm, voxel size = 1.25 × 1.25 × 1.25 mm, field of view = 200 mm, slice thickness = 1.25 mm, and flip angle = 80°), resulting in 243 volumes for the neutral movie and 250 volumes for the negative movie. The structural T1-weighted high-resolution scans were acquired using an MP2RAGE sequence (224 sagittal slices, echo time = 1.99 ms, repetition time = 4300 ms, inversion time 1 = 840 ms, inversion time 2 = 2370 ms, voxel size = 0.8 × 0.8 × 0.8 mm, flip angle = 5°/6° and slice thickness = 0.75 mm).

### 2.6 Imaging data preprocessing

The imaging data underwent preprocessing using fMRIPrep version 22.0.2 (Esteban et al., 2019), followed by post-preprocessing using XCP-D version 0.3.0 (Mehta et al., 2024). The preprocessing pipeline included slice-time correction, head-motion correction, co-registration between structural and functional images, normalization to MNI space, nuisance regression (i.e., the top 5 principal aCompCor components from white matter and cerebrospinal fluid compartments and the six motion parameters and their temporal derivatives), band-pass filtering within the 0.009–0.08 Hz, and smoothing of the images using a 4 mm kernel size. As part of the preprocessing, the first 3 dummy scans were discarded, leaving 240 volumes for the neutral movie and 247 volumes for the negative movie condition. Furthermore, region-of-interest (ROI) signal extraction was conducted based on the Schaefer 200-parcel cortical parcellation (Schaefer et al., 2018) and Tian 16-parcel subcortical parcellation (Tian et al., 2020), resulting in time series from 216 brain regions for each condition and for each participant. These 216 regions were assigned to 8 spatially independent brain networks (i.e., the visual network [VN], somatomotor network [SMN], dorsal attention network [DAN], ventral attention network [VAN], control network [CN], default mode network [DMN], limbic network [LN], and subcortical network [SUB]).

### 2.7 Inter-subject correlations

To test whether both neutral and negative movies elicit robust neural similarity across all participants, we calculated the participant-level intersubject correlation (ISC) by correlating the BOLD signal time series of each participant with the mean BOLD signal time series of the rest of participants (Fig. 1E). In other words, we assessed similarity by correlating each participant’s time course with a reference time course computed as the average of all other participants’ time courses. This procedure was conducted for each ROI and each participant, resulting in a 216 ROI × 62 participant-level ISC matrix. Furthermore, Pearson’s correlation and paired t-test were performed to compare the ISC between neutral and negative conditions, allowing us to estimate the similarity and variation of neural similarity between the two conditions.

The dyad-level ISC was calculated for each dyad (pair of participants) to indicate pairwise neural similarity during movie watching (Fig. 1D). To construct the ISC matrix, we computed Pearson’s correlation between the BOLD signal time series in each of the 216 brain regions for each pair of participants. With a total of 1891 unique dyads generated from the 62 participants included in the study, this procedure resulted in two 216 × 1891 ISC matrices separate for neutral and negative movies. Subsequently, the ISC matrices were transformed using Fisher’s *r-*to-*z* transformation to increase data normality.

### 2.8 Intersubject representational similarity analyses

In the current study, we conducted three IS-RSA analyses using linear mixed-effects (LME) models with crossed random effects to examine our hypotheses (G. Chen et al., 2017; Finn et al., 2020). To build these models, we utilized LME4 (version 1.1-32) and LMERTEST (version 3.1.3) in R. The LME models were advantageous as they explicitly modeled the nonindependence inherent in dyadic data by including crossed random effects for both members of each pair. This specification accounts for the shared variance contributed by participants who appear in multiple dyads, thereby effectively addressing the statistical dependencies between pairwise observations (Bernal-Rusiel et al., 2013; G. Chen et al., 2017).

To test the first hypothesis that resilience-related neural similarity may follow the AnnaK model, with high-resilience individuals exhibiting similar neural responses while low-resilience individuals having idiosyncratic neural responses, we performed IS-RSA with binarized resilience scores. Based on the median split of the overall resilience score (median = 67), participants were divided into a low-resilience group (N = 32, 14 females; mean resilience score ± SD: 57.22 ± 10.36, range: 22–67) and a high-resilience group (N = 30, 15 females; mean resilience score ± SD: 78.97 ± 7.92, range: 68–100, Fig. 3A). The chi-square test indicated no significant association between sex and resilience group (χ²(1) = 0.243, *p* = 0.622), suggesting that males and females were similarly distributed across the two resilience groups. This characterization resulted in 3 types of dyads among the 1891 unique pairs: (1) {high, high} for dyads where both participants were in the high-resilience group; (2) {high, low} for dyads with one high-resilience and one low-resilience participant; and (3) {low, low} for dyads with both participants in the low-resilience group. Subsequently, we conducted the first IS-RSA by constructing LME models, incorporating random effects of participants within each dyad. The model included the dyad types (i.e., {high, high}, {high, low}, and {low, low}) as fixed effects and the dyad-level ISC for a given brain region as the dependent variable, as presented in the following formula:

$$S_{ij}=\;\sum_{g\in \left\{HH,HL,LL\right\}}\mu_{g}\cdot I\left(dyad\left(i,j\right)=g\right)+\;\alpha_{i}+\alpha_{j}+\varepsilon_{ij},$$ (1)

where $S_{ij}$ is the dyad-level ISC value for the dyad formed by subjects i and j; $\mu_{g}$ denotes the fixed-effect mean ISC for dyad type g∈{HH,HL,LL}; I(dyad(i,j)=g) is an indicator function equal to 1 if dyad (i,j) belongs to group g, and 0 otherwise; $\alpha_{i}$ and $\alpha_{j}$ are the random subject intercepts for subjects i and j, respectively, modeling the non-independence of subjects across dyads; $\varepsilon_{ij}$ is the residual error term. A planned-contrast analysis was then used to examine and compare the neural similarity among the three types of dyads. The planned contrasts were defined between three dyad types: {high, high} versus {low, low}, {high, high} versus {high, low}, and {high, low} versus {low, low}. Estimated marginal means and contrasts were obtained using the *emmeans* package in R. For each contrast, we assessed the estimated coefficient (*β*), which quantifies the estimated marginal mean difference for each planned contrast. As separate contrast analyses were conducted for each brain region, a false discovery rate (FDR) correction was applied to account for multiple comparisons.

To explore the first hypothesis in a greater detail, we further performed the second IS-RSA with resilience similarity as the independent variable. Based on the AnnaK principle (Ohad & Yeshurun, 2023), the resilience similarity was defined by the minimum resilience score for each dyad. Thus, only pairs of high-resilience individuals showed high similarity (i.e., high-resilience individuals are alike), whereas pairs of low resilience or mixed pairs (a pair consists of one individual with low resilience and one with high resilience) were consistently characterized by low similarity (i.e., low-resilience individuals differ from their peers as well as high-resilience individuals) (Fig. 3C). To ensure the normality of the data, a log-transform was further applied to resilience similarity scores. This allowed us to test whether resilience similarity could predict the neural similarity observed in dyads. The LME model was specified as follows:

$$S_{ij}=\beta_{0}+\beta_{1}\cdot R_{ij}+\alpha_{i}+\alpha_{j}+\varepsilon_{ij},$$ (2)

where i and j  denote the two subjects forming a dyad, $S_{ij}$ represents the dyad-level ISC between the pair of subjects i and $j,\;\;\beta_{0}$ denotes the intercept, $R_{ij}$ denotes the resilience similarity between subjects i and j, $\beta_{1}$ denotes the fixed-effect coefficient of resilience similarity, $\alpha_{i}$ and $\alpha_{j}$ are the random subject intercepts for subjects i and j, respectively, and $\varepsilon_{ij}$ is the residual error term. By doing so, we aimed to gain deeper insights into how the similarity in resilience scores of dyads relates to the extent of neural similarity observed in their brain responses.

Lastly, we further examined our second hypothesis and investigated whether IU is associated with resilience-related neural similarity. The product of the IU scores within each dyad was normalized to a 0–1 range, resulting in joint intolerance of uncertainty score (JIUS), indicating both the similarity of IU between two individuals within the dyads and the absolute position of the dyad members on the IU spectrum (i.e., the level of IU). We performed the third IS-RSA, which investigated the effect of IU by introducing the interaction effect between resilience similarity and JIUS, as presented in the following formula:

$$S_{ij}=\beta_{0}+\beta_{1}\cdot R_{ij}+\beta_{2}\cdot J_{ij}+\beta_{3}\cdot R_{ij}\times J_{ij}+\alpha_{i}+\alpha_{j}+\varepsilon_{ij}.$$ (3)

Here, $J_{ij}$ denotes the JIUS between subjects i and j, $\beta_{2}$ denotes the effect of JIUS, $\beta_{3}$ denotes the effect of interaction between JIUS and resilience similarity; all other terms follow the definitions provided in Formula 2. For each IS-RSA, FDR correction with *p* < 0.05 was applied to control for multiple comparisons.

### 2.9 Validation analyses

Given the similarity in neural responses is associated with demographic factors (Finn et al., 2020; Nummenmaa et al., 2018), we validated our results and accounted for the potential impact of demographic similarity on neural similarity. Because head motion was minimal in our sample (mean FD = 0.11 mm for the neutral movie and 0.12 mm for the negative movie), strict motion exclusion criteria (average FD > 0.25 mm) had already been applied, and motion did not correlate with any of the key behavioral variables (neutral movie: *r* = 0.216, *p* = 0.093 for resilience; *r* = 0.177, *p* = 0.167 for IU; negative movie: *r* = 0.146, *p* = 0.257 for resilience; *r* = 0.156, *p* = 0.224 for IU), motion was not included as covariates in validation analyses.

In this analysis, we added the similarity of age, sex, and education as covariates into the LME models to assess the robustness of our findings. For sex, we employed an indicator variable to represent the sex similarity of each dyad, with 1 indicating the same sex and 0 indicating different sexes. Regarding age, we calculated the L1 distance between the ages of each pair of participants. The L1 distance was then normalized to a range between 0 and 1, and the resulting value was subtracted from 1 to obtain a measure indicating the similarity in age. A similar procedure was conducted to obtain similarity in education.

Furthermore, to mitigate the concern about using the median split for grouping, which may treat individuals with very similar scores as belonging to different groups, we adopted an alternative grouping approach based on the lower and upper quartiles of the resilience scores’ distribution. This procedure yielded two groups: a low-resilience group (N = 16, 7 females; mean = 50.56 ± 11.09, range: 22–60) and a high-resilience group (N = 16, 8 females; mean = 84.88 ± 5.78, range: 80–100). Although the resulting group sizes are relatively small, this quartile-based comparison serves as a meaningful robustness check and provides additional validation for the pattern observed in our first IS-RSA.

Lastly, we conducted control analyses to investigate neural similarity related to depression, anxiety, and stress. The same analytic procedure used in the primary IS-RSA was applied to binarized depression and anxiety scores (based on the clinical cutoff of 7 on the HADS, Wu et al., 2021), as well as to binarized stress scores (based on a median split). These analyses help clarify that the relationship we reported between resilience and neural similarity is unlikely to be explained by these alternative behavioral dimensions, even though they are highly correlated with resilience.

## 3 Results

### 3.1 Movies effectively trigger corresponding emotional experiences

**Fig. 2.**
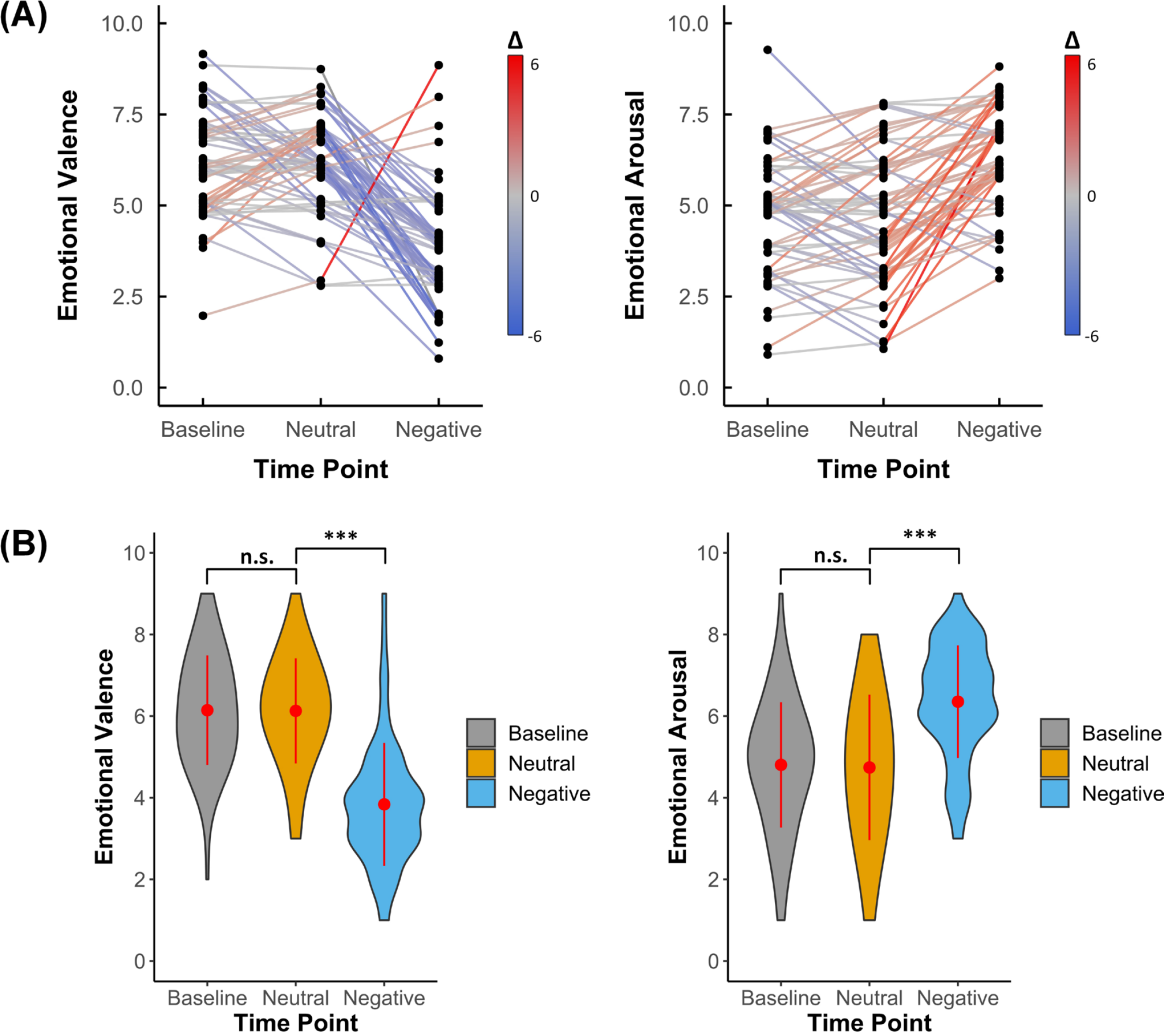
Emotional valence and arousal ratings before and after viewing the neutral and negative movies (participants N = 62). (A) The emotional valence and arousal ratings for the before (baseline) and after each movie. The colors of the lines indicate changes in individual responses for the emotional ratings. (B) The violin plots of the emotional valence and arousal ratings. No significant differences were observed between baseline and after viewing the neutral movie in emotional valence, and the same results were obtained for emotional arousal. However, a significant decrease in emotional valence and a significant increase in emotional arousal were observed after participants watched the negative movie. The red points within the violin plots indicate the mean value of observation, and the red lines indicate the standard deviations. n.s.: not significant; ***: *p* < 0.001.

Participants reported their emotional valence and arousal levels before and after each movie clip. After watching the neutral movie, no significant changes were observed in emotional valence (*t* = 0.095, *df* = 61, *p* = 0.925, *d* = 0.012) and arousal (*t* = 0.375, *df* = 61, *p* = 0.709, *d* = 0.048) compared with the baseline, prior to watching the movie. However, after watching the negative movie, compared with the neutral clip, a significant decrease in valence (*t* = -9.193, *df* = 61, *p* < 0.001, *d* = 1.167) and an increase in arousal (*t* = 7.298, *df* = 61, *p* < 0.001, *d* = 0.927) were observed (Fig. 2). These results demonstrate the validity of our naturalistic stimuli. Furthermore, no significant correlations were found between resilience scores and emotion ratings (all *p*’s > 0.075), nor resilience score and alterations of emotional status (all *p*’s > 0.1).

We additionally examined sex differences in behavioral measures in our pilot sample presented in the Supplementary Table S9. In our main sample, sex differences were generally minimal across both emotional ratings and psychological measures. Male and female participants did not differ in valence or arousal across the baseline, neutral (*Pottery*), and negative (*Curve*) clips, indicating highly comparable emotional responses regarding the movies across sexes. Moreover, no significant sex differences were observed in our primary variables—resilience and IU—or for related measures, including stress, depression, and most DERS dimensions (Supplementary Table S10). Although a few isolated measures (such as anxiety and select DERS subscales) showed significant sex differences, these effects may merit further examination in future work but are beyond the scope of the current study. Together, these results indicate that sex did not significantly influence either emotional ratings or the primary variables of interest in our main sample.

### 3.2 Low resilience is associated with low emotional well-being

We investigated the relationship between resilience and various mental health indicators. Negative correlations between resilience and IU (*r* = -0.395, *p* = 0.001), anxiety (*r* = -0.314, *p* = 0.013), depression (*r* = -0.306, *p* = 0.016), and perceived stress (*r* = -0.489, *p* < 0.001) were found. Moreover, negative associations were found between resilience and multiple DERS subscales, including nonacceptance of emotional response (*r* = -0.401, *p* = 0.001), lack of emotional awareness (*r* = -0.423, *p* < 0.001), limited access to emotion regulation strategies (*r* = -0.377, *p* = 0.002), and lack of emotional clarity (*r* = -0.404, *p* = 0.001). Full details of zero-order correlations between resilience, IU, and related variables, including depression, anxiety, perceived stress, and emotion regulation difficulties are presented in Table 1. In summary, our behavioral data suggest that individuals with low resilience may show a higher risk of mood disorders, signifying their vulnerability to different emotional and cognitive dysfunctions.

**Table 1.** Pearson’s correlations between interested variables (N = 62).

	1	2	3	4	5	6	7	8	9	10
1. Resilience	1									
2. IU	-.395^**^	1								
3. Anxiety	-.314^*^	.537^**^	1							
4. Depression	-.306^*^	.373^**^	.449^**^	1						
5. Stress	-.489^**^	.628^**^	.588^**^	.531^**^	1					
6. DERS										
DERS-1	-.401^**^	.287^*^	.421^**^	.197	.422^**^	1				
DERS-2	-.248	.275^*^	.220	.396^**^	.384^**^	.180	1			
DERS-3	-.162	.382^**^	.191	.315^*^	.403^**^	.178	.319^**^	1		
DERS-4	-.423^**^	.112	.060	.313^*^	.276^*^	.149	.017	.246	1	
DERS-5	-.377^**^	.353^**^	.278^*^	.434^**^	.543^**^	.507^**^	.414^**^	.460^**^	.359^**^	1
DERS-6	-.404^**^	.300^*^	.180	.390^**^	.436^**^	.300^**^	.172	-.018	.599^**^	.457^**^

The correlation analysis was performed to illustrate the relationship between resilience, intolerance of uncertainty (IU), anxiety, depression, and difficulties in emotion regulation. Resilience was measured by the Connor–Davidson Resilience Scale. Intolerance of uncertainty (IU) was assessed by the Intolerance of Uncertainty Scale. Anxiety and depression were measured by the Hospital Anxiety and Depression Scale. Stress was measured with the Perceived Stress Scale. Emotion regulation problems were evaluated by the Difficulties in Emotion Regulation Scale (DERS). The DER subscales from 1 to 6 represent the following difficulties in emotion regulation: 1—Nonacceptance of emotional responses; 2—Difficulty engaging in goal-directed behavior; 3—Impulse control difficulties; 4—Lack of emotional awareness; 5—Limited access to emotion regulation strategies; 6—Lack of emotional clarity. ^*^: *p* < 0.05; ^**^: *p* < 0.01.

### 3.3 Negative movie elicits stronger neural similarity

To assess the robustness of neural similarity elicited by both neutral and negative movies across all participants, we computed the participant-level ISC for each brain region.

**Fig. 3.**
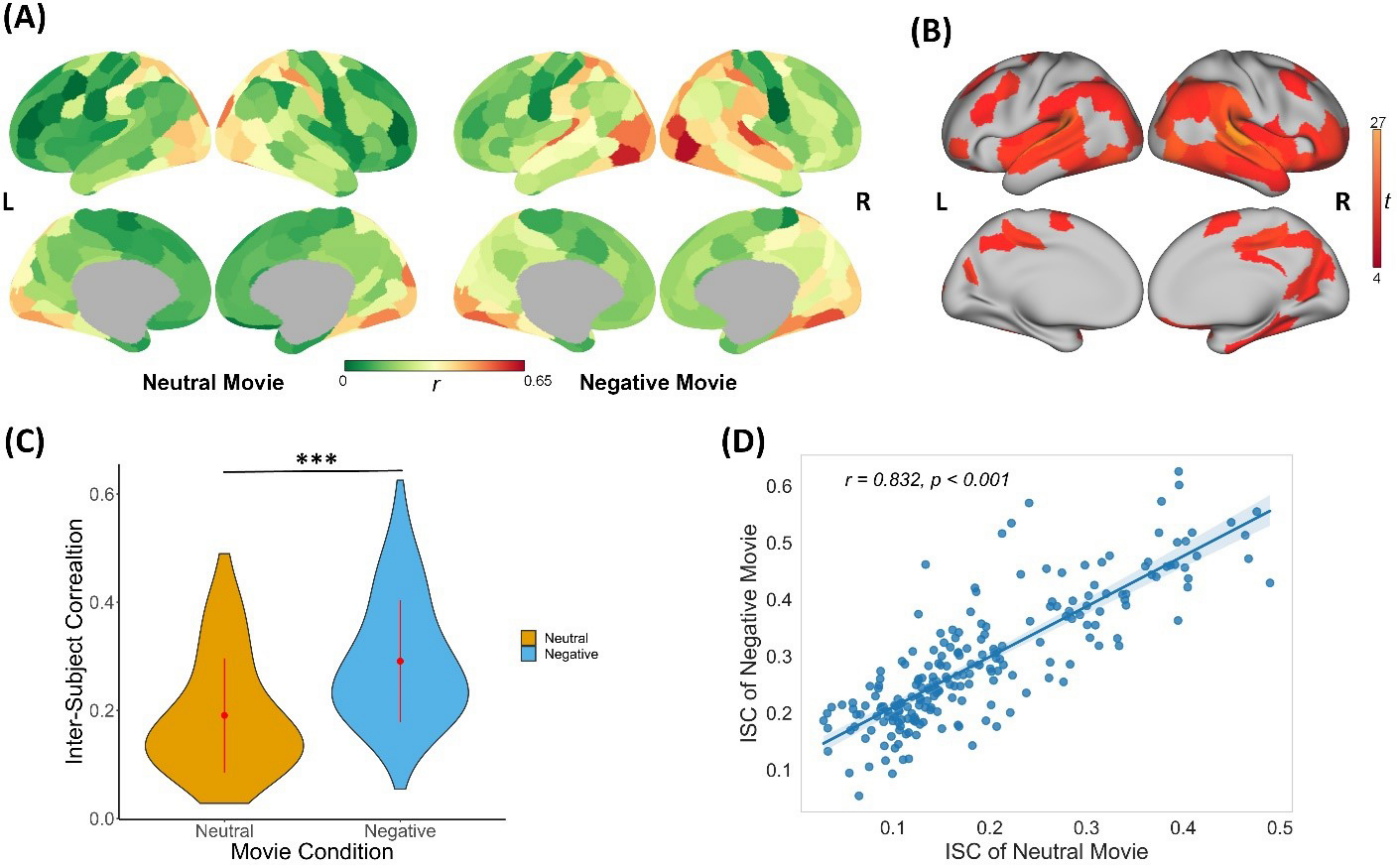
Participant-level inter-subject correlation (ISC) of the neutral and negative movies. (A) The participant-level ISC maps for neutral and negative movies. (B) Results of the paired t-test (FDR corrected) comparison between ISC of neutral and negative movie. The higher *t*-values indicate higher ISC elicited during the negative than during the neutral movie. (C) The negative movie induced overall greater neural similarity than the neutral movie. The red points within the violin plots indicate the mean value of the observation, and the red lines indicate the standard deviations. (D) The spatial distribution of ISC maps of two conditions is highly consistent. Each data point represents one brain area. The shaded zone represents 95% Confidence Interval (CI). ***: *p* < 0.001.

The results indicated an overall pattern of neural similarity for both movies, with the most pronounced effects in auditory and visual brain areas (Fig. 3A). However, we observed significant differences in participant-level ISC between the two movie conditions. Higher ISC values were evident in the temporal lobe, prefrontal cortex, posterior cingulate cortex, precuneus, and subcortical areas (such as the hippocampus, amygdala, and caudate) during the negative movie as compared with the neutral movie (Fig. 3B; Supplementary Table S2). Moreover, participants exhibited higher levels of global neural similarity during the negative condition than during the neutral condition (mean ISC of the neutral movie = 0.191, mean ISC of the negative movie = 0.291, *t* = 23.255, *df* = 217, *p* < 0.001, *d* = 1.582, Fig. 3C). Additionally, the spatial distribution of neural similarity was highly correlated across the two conditions (*r* = 0.832, *p* < 0.001, Fig. 3D), suggesting a stable functional organization in response to naturalistic stimuli regardless of the emotional content of the movies. In summary, the negative movie induced stronger neural similarity than the neutral movie, especially in areas that are implicated in affective function (e.g., the insula and amygdala), social cognition (e.g., the prefrontal cortex and inferior parietal lobe), and memory retrieval (e.g., the posterior cingulate cortex and hippocampus).

### 3.4 Individuals with higher resilience exhibited greater neural similarity

To test the first hypothesis regarding whether individuals with higher resilience scores exhibit higher neural similarity during movie watching, we conducted a comparison between high- and low-resilience individuals using the IS-RSA with dyad-level ISC.

**Fig. 4.**
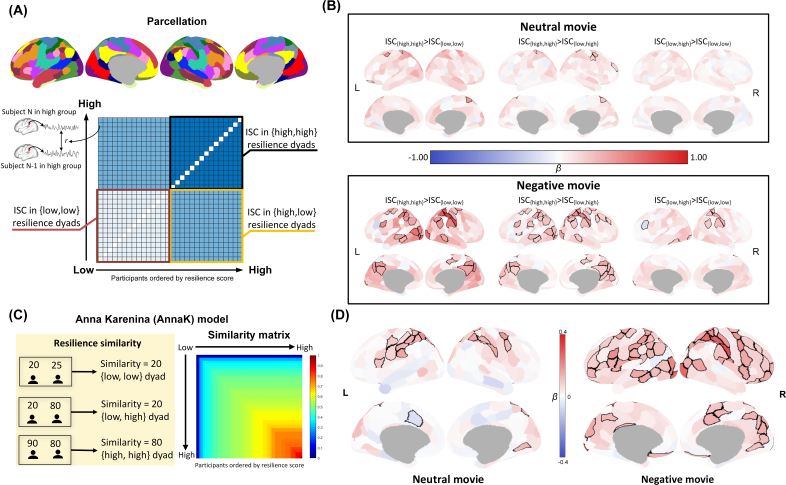
Results of dyad-level analyses on the relationship between resilience and neural similarity. (A) All dyads were classified into three types, {high, high}, {high, low}, and {low, low}, based on the resilience levels of their respective members. The dyad-level Inter-Subject Correlation (ISC) was then calculated by correlating the BOLD signal time series of the two participants within each dyad. This allowed for the creation of a dyad-level ISC matrix for each brain region. (B) The results of the first IS-RSA and the planned contrasts comparing ISC across the three dyad types. The β-value reflects the estimated mean difference in ISC between the dyad types specified in each contrast. Regions showing significant differences between the two dyad types after false discovery rate (FDR) correction are outlined in black. (C) The calculation of resilience similarity in accordance with Anna Karenina model. (D) The results of the second IS-RSA that link resilience similarity and neural similarity. The β is the standardized regression coefficient relating resilience similarity to ISC. Regions with significant associations between their neural similarity and resilience similarity after FDR correction are outlined in black. L, left hemisphere; R, right hemisphere.

Participants were categorized into two groups based on their resilience scores, resulting in three types of dyads based on the resilience group membership of the pairs: {high, high}, {high, low}, and {low, low}. During the neutral movie, higher ISCs were found in {high, high} dyads than in the {low, low} dyads in some regions, encompassing the postcentral gyrus, frontal eye fields, and ventral prefrontal cortex (Fig. 4B; Supplementary Table S3). We also found higher ISCs in the {high, high} dyads than in the {high, low} dyads in the postcentral gyrus, lateral prefrontal cortex, and frontal eye fields. No significant results were found in the comparison between {high, low} and {low, low} dyads.

Similar patterns were observed in the negative movie, but the group differences in ISC were found across a broader set of brain areas in all contrasts (Fig. 4B; Supplementary Table S4). Specifically, higher ISCs in multiple brain regions that mainly anchored in the DAN, CN, and DMN were found in the {high, high} dyads than in either {low, low} or {high, low} dyads. Moreover, ISCs in several brain regions including the visual cortex, inferior parietal lobe, lateral prefrontal cortex, and postcentral gyrus showed differences between {high, low} dyads and {low, low} dyads.

Collectively, our findings indicate greater neural similarity in dyads of high-resilience pairs {high, high} than in dyads of low-resilience pairs {low, low} or mixed pairs {high, low} during viewing both neutral and negative movies, suggesting that individuals with high resilience exhibit more consistent neural responses than their peers. In contrast, individuals with low resilience display more diverse neural responses among themselves.

To further investigate the relationship between neural similarity and resilience, we performed a second IS-RSA utilizing resilience similarity to predict dyad-level ISC, which provides a more fine-grained examination of the resilience–ISC association and can validate our results from the first IS-RSA. The results revealed that resilience similarity significantly predicted neural similarity in several brain regions, including the postcentral gyrus, frontal eye fields, and inferior parietal area, while participants were viewing the neutral movie (Fig. 4D; Supplementary Table S5). Notably, many of these observed brain regions are core components of the DAN. For the negative movie, we found a positive association between resilience similarity and neural similarity in areas of multiple brain networks including the DMN (e.g., the inferior parietal lobe, posterior cingulate cortex, and precuneus), DAN (e.g., the frontal eye field, postcentral gyrus, superior parietal lobule), CN (e.g., the lateral prefrontal cortex and intraparietal sulcus), SMN, and VN (Fig. 4D; Supplementary Table S6). Consistent with our primary results based on binarized resilience categories, greater neural similarity in individuals with higher resilience was found.

### 3.5 Intolerance of uncertainty is associated with resilience-related neural similarity

**Fig. 5.**
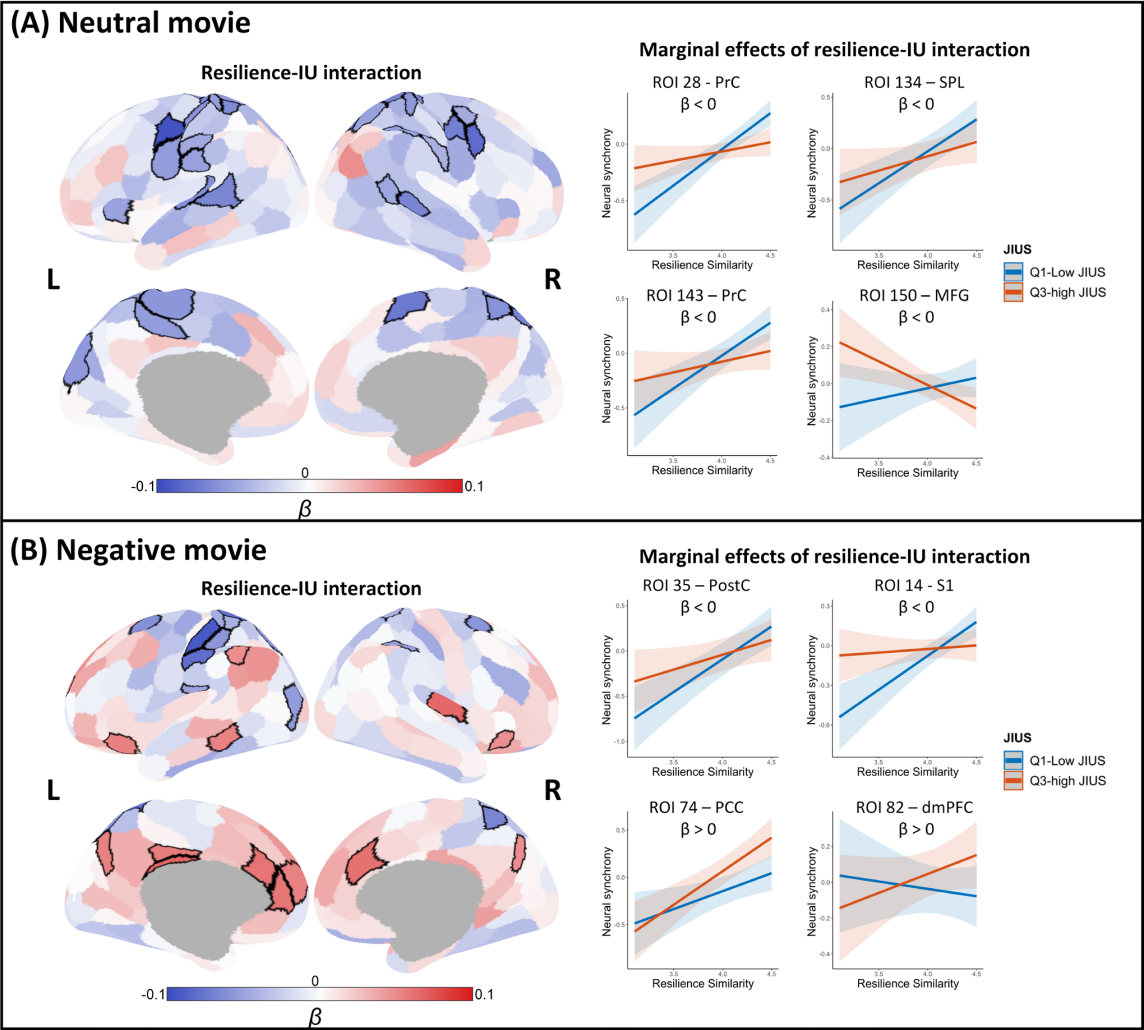
Modulation effects of intolerance of uncertainty (IU) on resilience-related neural similarity. (A) The results of the neutral movie. The brain maps illustrate that neural similarity in brain areas, in blue and outlined in black, was dampened by the interaction between IU and resilience. Estimated marginal effects for neural similarity by joint intolerance of uncertainty score (JIUS) indicated increased IU attenuated the resilience-related neural similarity. Four regions (bilateral precentral gyrus [ROI 28 and 143 – PrC], left superior parietal lobe [ROI 34 – SPL], and left middle frontal gyrus [ROI 150 – MFG]) that exhibited a large modulation effect of IU are presented as examples here. (B) The results of the negative movie. The brain maps illustrate that neural similarity in brain areas, in blue and outlined in black, was dampened by the interaction between IU and resilience, and brain areas, in red and outlined in black, were enhanced by the IU–resilience interaction. Four regions (left postcentral gyrus [ROI 35 – PostC], left primary somatosensory cortex [ROI 14 – S1], left posterior cingulate cortex [ROI 74 – PCC], and left dorsomedial prefrontal cortex [ROI 82 – dmPFC]) that exhibited a large modulation effect of IU are presented as examples to demonstrate the estimated marginal effects. Q1 is the lower quartile and Q3 is the upper quartile of JIUS. Shaded areas represent 95% CIs.

Lastly, we tested the impact of IU on resilience-related neural similarity with the hypothesis that individuals with higher IU exhibit lower resilience-related neural similarity, particularly in the DAN. As hypothesized, our findings showed that increased IU was linked to resilience-related neural similarity in regions associated with attention and perception (i.e., DAN subregions including the superior parietal lobe and postcentral gyrus). Specifically, individuals with high IU and high resilience, compared with those with low IU and high resilience, exhibited reduced neural similarity in areas involved in attentional engagement, visuospatial processing, and sensorimotor function. We also found that the resilience–IU interaction negatively predicted neural similarity during the neutral movie in the regions that are functionally associated with sensorimotor (e.g., the precentral gyrus), spatial attention (e.g., the intraparietal sulcus and superior parietal lobule), and auditory (e.g., the superior temporal gyrus) functions (Fig. 5A; Supplementary Table S7). For negative movie, the attenuation of resilience-related neural similarity by IU was observed in motor- and attention-related regions (e.g., primary sensorimotor cortex, postcentral gyrus, and intraparietal sulcus) (Fig. 5B; Supplementary Table S8). Moreover, we found that the resilience–IU interaction positively predicted ISC in the dorsomedial prefrontal cortex, ventral prefrontal cortex, posterior cingulate cortex, precuneus, inferior parietal lobe, and insula during the negative movie (Fig. 5B; Supplementary Table S8).

### 3.6 Validation results

**Fig. 6.**
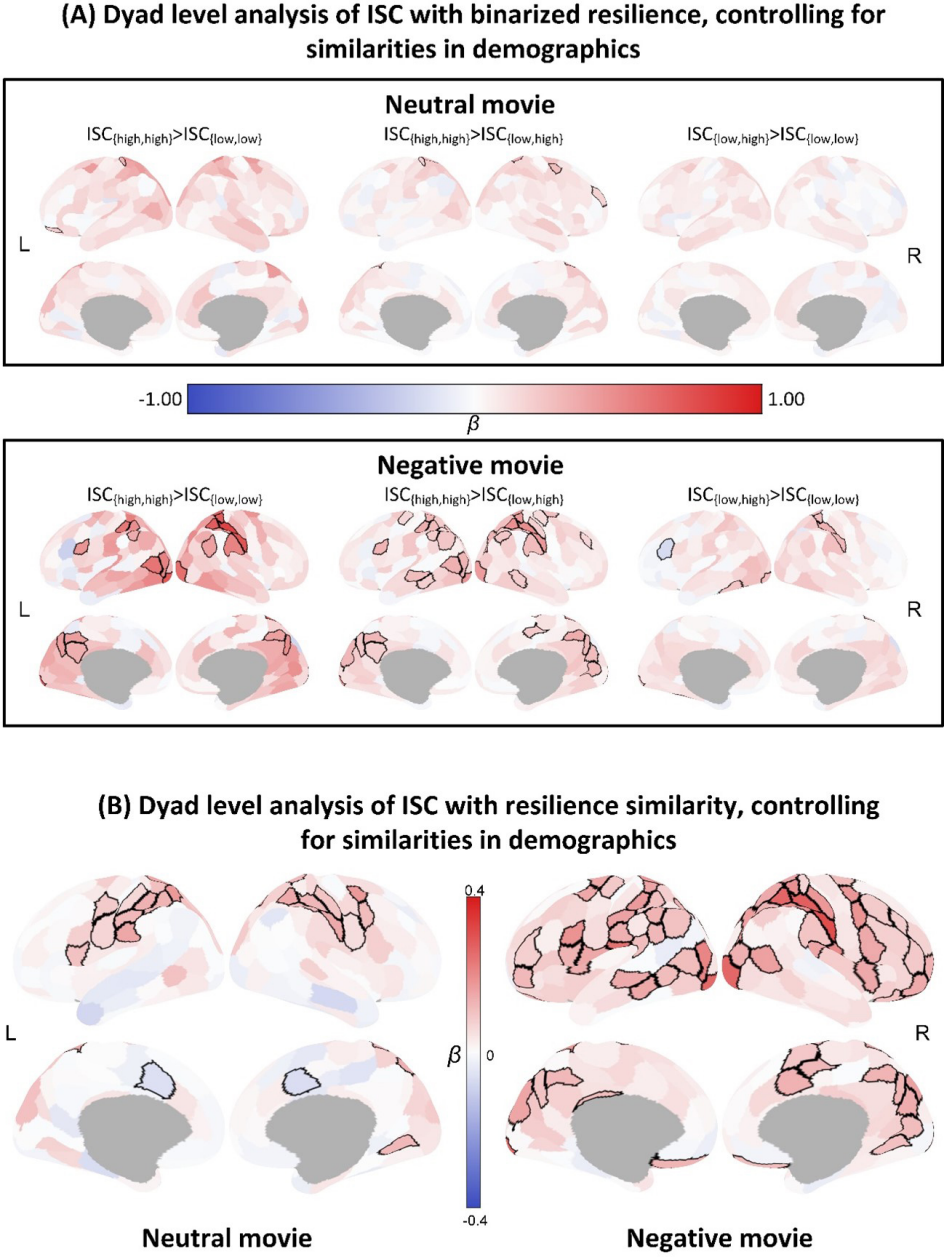
Results of IS-RSA validation where demographic similarities were controlled. (A) The results of IS-RSA and the planned contrasts comparing ISC across the three dyad types, while controlling demographics including age, sex, and education. Similar patterns to our main findings were observed even after controlling for confounding variables. In the neutral condition, higher neural similarity was observed in {high, high} dyads than in {low, low} and {low, high} dyads. These patterns were even stronger in the negative condition. (B) Association of neural similarity with resilience similarity while controlling demographics including age, sex, and education. We identified similar brain regions that showed resilience-related neural similarity as our main findings. Regions with significant differences or associations are outlined in black. FDR corrections with a significance threshold of *p* < 0.05 were applied to address the issue of multiple comparisons.

The validation analysis, which further accounted for demographic similarity, produced results that closely aligned with our primary findings (Fig. 6). Therefore, the observed neural similarity is mainly explained by the similarities in participants’ resilience levels instead of the similarity in demographics including age, sex, and education. We further repeated the IS-RSA using a quartile-based binarization of resilience scores and again observed the same pattern: {high, high} dyads showed significantly higher neural similarity than both {low, low} and {low, high} dyads across both movie conditions (Supplementary Fig. S2), demonstrating the robustness of our main findings. Moreover, validation analyses using additional behavioral measures—including anxiety, depression, and stress—revealed patterns distinct from resilience (Supplementary Fig. S3), further supporting that our main effects are specific to resilience rather than other behavioral dimensions.

### 3.7 Sex difference in behavioral measures and neural similarity

We conducted additional post hoc analyses to assess potential sex differences in behavioral measures and neural similarity. The results of sex differences in behavioral measures are presented in Supplementary Table S9. In our main samples, sex differences were generally minimal across both emotional ratings and psychological measures. Male and female participants did not differ in valence or arousal across the baseline, neutral (*Pottery*), and negative (*Curve*) clips, indicating highly comparable emotional responses regarding the movies across sexes. Moreover, no significant sex differences were observed in our primary variables—resilience and IU—or for related measures, including stress, depression, and most DERS dimensions. Although a few isolated measures (such as anxiety and select DERS subscales) showed significant sex differences, these effects may merit further examination in future work but are beyond the scope of the current study. Together, these results indicate that sex did not significantly influence either emotional ratings or primary variables of interest in our main sample.

We subsequently evaluated sex differences in participant-level ISC (Supplementary Fig. S4A, B). Males demonstrated lower global ISC than females for both the neutral (*t* = -2.039, *p* = 0.046, *d* = -0.519) and negative movies (*t* = -2.542, *p* = 0.014, *d* = -0.647). Further ROI-level analyses with FDR correction revealed that during the negative movie, females exhibited higher ISC in the left temporal pole (*t* = -3.394, *p* = 0.045), left precuneus (*t* = –3.949, *p* = 0.015), right ventrolateral prefrontal cortex (*t* = –3.713, *p* = 0.025), right intraparietal sulcus (*t* = –3.503, *p* = 0.038), right inferior parietal lobule (*t* = –4.157, *p* = 0.015), and right dorsolateral prefrontal cortex (*t* = –4.032, *p* = 0.015), while no significant sex differences were observed in subcortical regions. Moreover, no ROI-level effects survived FDR correction for the neutral movie condition. We also separately examined the spatial correspondence of ISC strength for the neutral and negative movies within the male and female groups, and the results indicated that the spatial distribution of ISC was consistent across sexes (Supplementary Fig. S4C). These results provide preliminary evidence that while sex-related factors might influence the overall strength of neural similarity, they may not substantially alter the functional brain organization during naturalistic viewing.

Lastly, we computed dyad-level ISC and performed linear mixed effects and planned contrast analyses to test whether neural similarity differed as a function of dyadic sex composition (Supplementary Fig. S4D). As in our main analysis, three types of dyads (i.e., {Female, Female}, {Female, Male}, {Male, Male}) among the 1891 unique pairs were produced. LME models and planned contrasts were then applied to these groups. Since these analytical procedures closely parallel those described in the main text, we provide only a summary here to avoid unnecessary repetition. We found greater neural similarities in {Female, Female} dyads than in dyads of {Female, Male} and {Male, Male}. Intriguingly, neural similarity among {Female, Male} dyads was lower than that observed in {Male, Male} dyads, which suggests that the variability in brain responses among men may be greater than the variability between men and women. These effects are more pronounced in the negative movie. The overall pattern suggests that sex differences may follow the AnnaK model, such that females display more convergent and homogeneous brain responses, whereas males exhibit more heterogeneous neural patterns. It is important to note that in our main analyses, sex was evenly distributed across the high- and low-resilience groups, and adjusting for sex did not change the primary findings. Thus, the observed sex differences are unlikely to influence the main conclusions.

## 4 Discussion

Despite growing recognition of the role of resilience in mental well-being, our understanding of neurobiological circuits underlying resilience remains limited. Neuroimaging studies have yet to clarify whether resilience operates as a dynamic process actively engaged in stress responses or as a static trait that functions passively. Distinguishing between these perspectives is crucial, as it determines whether resilience should be conceptualized as a modifiable process that can be strengthened through interventions or as a stable characteristic that primarily reflects individual differences (Stainton et al., 2019; Vella & Pai, 2019). By using movie-fMRI and IS-RSA, our findings revealed the convergent pattern of neural similarity in brain areas involved in attention modulation, emotion regulation, and cognitive control, which were associated with resilience. We also showed that IU, a trait related to heightened sensitivity to ambiguity and unpredictable situations, was associated with resilience-related neural similarity. Our results provide an important contribution to understanding how individuals with varying levels of resilience perceive and process naturalistic stimuli.

We identified a convergent pattern of neural similarity associated with psychological resilience, observed in both IS-RSA based on group stratification by resilience levels and IS-RSA using resilience similarity estimated from continuous resilience scores. Specifically, individuals with high resilience exhibited similar neural responses, while those with low resilience displayed divergent neural responses to external stimuli. This pattern concords with the AnnaK model, commonly employed to elucidate the vulnerability of multi-component systems (Finn et al., 2020; Iyer et al., 2024; Zaneveld et al., 2017). According to this model, system dysfunction occurs when deficits exist in any of the essential components. In the context of the current study, we suggest that resilience-related responses could be described as a system comprising multiple emotional processes. To maintain balanced mental states after exposure to negative stimuli, adaptive responses from all components of emotional processing, encompassing emotion perception, evaluation, integration, and regulation, are critical (Foa et al., 2006; Gloria & Steinhardt, 2016). We speculate that resilient individuals sustain adaptability throughout these processes, ultimately leading to successful responses to and regulation of negative events. Conversely, individuals characterized as low in resilience may generally exhibit reduced adaptive capacity, yet the underlying reasons for this outcome can differ, reflecting dysfunctions in distinct components of the resilience response. Our behavioral results, indicating negative correlations between resilience and various emotion regulation difficulties, further support the notion that resilient individuals can effectively maintain their mental health (Ungar & Theron, 2020), whereas individuals with low resilience exhibit different emotion regulation difficulties (Kim et al., 2021; Poole et al., 2017) and are more susceptible to various mental disorders (Bhatnagar, 2021; Herrman et al., 2011). Thus, high-resilience individuals can maintain high mental well-being, which is reflected in their more similar neural responses to one another, suggesting shared adaptive processes in response to external stimuli. In contrast, low-resilience individuals exhibited greater variability in neural responses, indicating that although all are characterized by low resilience, the underlying causes may differ, reflecting dysfunctions in distinct components of the resilience response.

Resilience-related neural similarity was specifically observed in regions anchored in the DAN. During naturalistic movie watching, top–down attention enhances focus from a specific perspective, aids in gathering relevant information, and enables the spread of information across brain networks (Regev et al., 2019). Consequently, the neural similarity in the DAN may reflect the aligned neural encoding of external stimuli (Nummenmaa et al., 2012), implying a shared perspective among participants during movie watching. In the negative movie clip, the resilience-related neural similarity extended to other brain networks, including the DMN and CN. Empirical evidence suggests that the high neural similarity of DMN and CN signifies shared narrative interpretation among audiences (Nguyen et al., 2019). The DMN is reported as a key network in social cognition, with one of its core functions being linking with the actions of other social agents and characterization of interpersonal interactions (Yeshurun et al., 2021). Previous studies have found that the increased DMN functional coupling between speaker and listener predicts a more similar understanding of the story between them (Silbert et al., 2014). The CN is implicated in cognitive control and emotion regulation, which are fundamental for facilitating the understanding of events (Morawetz et al., 2020; Niendam et al., 2012). High neural similarity within these two networks suggests a similarity in their interpretation of the movie among resilient individuals, possibly stemming from their similarity in emotion regulation and evaluation of external stimuli when processing negative events. Based on these findings, we speculate that resilience reflects the overall adaptability of emotional functions, requiring coordination among multiple networks, primarily including the DAN, DMN, and CN.

The current findings align with previous research indicating that resilience involves multiple brain networks and is closely intertwined with emotional processing (Li et al., 2024). Building upon these neuroimaging discoveries and recognizing the intricate nature of emotional processes in response to negative events, it is important to consider resilience as an ongoing and dynamic neural process that encompasses dimensions of emotion and attentional mechanisms (Scherer & Moors, 2019). This dynamic characteristic is well manifested while participants engage in processing naturalistic stimuli that unfold over time, thus providing a methodological advantage of uncovering neural correlates associated with resilience that are dismissed using traditional task-based designs. While our study did not specifically focus on emotional recognition, it extends previous hypotheses about emotions, such as the constructionist approach (Lindquist et al., 2012; Xu et al., 2021), to naturalistic movies. These theories posit that the evaluation and attentional orientation of emotionally valenced naturalistic stimuli rely on the coordinated engagement of multiple brain networks (Kringelbach et al., 2023; Nummenmaa et al., 2023). Therefore, future studies should incorporate more naturalistic movies to test hypotheses and models of emotions, aiming to examine the generalizability of existing models to more dynamic and naturalistic stimuli.

Additionally, we made a noteworthy discovery that IU plays a role in the relationship between resilience and neural similarity. Consistent evidence from empirical studies across diverse samples has demonstrated a negative association between IU and resilience (Di Trani et al., 2021; Panzeri et al., 2021; T. Wang et al., 2023). In line with this evidence, we found that higher IU was associated with reduced resilience-related neural similarity, predominantly within DAN subregions across both movie conditions. This pattern aligns with findings that IU is linked to poorer attentional inhibition—an ability largely governed by the DAN—which may diminish goal-directed attentional engagement with external stimuli and increase variability in DAN activity (Morriss & McSorley, 2019). Beyond the DAN, we also observed similar IU-related modulation in regions involved in processing motor, visual, and auditory information. The broad modulation effects suggest that individuals with higher IU may engage in more variable attention control and processing of sensory inputs, ultimately manifesting as reduced resilience-related neural similarity. Importantly, the modulatory influence of IU aligns with the AnnaK principle, which emphasizes the importance of intact components in a system to maintain proper functioning. In other words, IU may dampen resilience response by impairing the attention functions critical for gating external information and gathering emotional cues. Moreover, the IU-related stronger positive association between resilience similarity and neural similarity in multiple brain regions—including the prefrontal cortex, posterior cingulate cortex, precuneus, inferior parietal lobe, and insula—was observed only during the negative movie. Previous evidence suggests that these areas play key roles in emotion recognition and regulation, as well as in generating shared interpretations of events (Morawetz et al., 2020; Yeshurun et al., 2021). Our findings may, therefore, suggest that increased IU promotes the formation of a coherent, clear, and certain interpretation and understanding of emotional narratives in resilient individuals, which may allow them to avoid negative feelings stemming from the uncertainty associated with novel emotional stimuli.

Several limitations should be noted when interpreting our findings. First, resilience was assessed using a self-report questionnaire. Although widely used (Didriksen et al., 2025; Gonzalez et al., 2016; Long et al., 2019; Miyagi et al., 2020; Pan et al., 2024), this measure primarily captures trait-level resilience and may not fully reflect its dynamic and multifaceted nature. In addition, deriving neural correlates from questionnaire-based stratification risks conflating the measurement with the outcome. The use of a median split further introduces limitations, especially without a validated clinical cutoff, as it may artificially separate individuals with similar scores. Future studies could adopt more comprehensive approaches to assess resilience and perform group stratification, such as incorporating additional pathological traits and applying data-driven clustering based on behavioral measures (Kaldewaij et al., 2021). Another promising direction is to stratify individuals according to their neural responses to naturalistic stimuli and then examine whether these brain-based groupings converge with questionnaire-based resilience scores (Bi et al., 2024; Y. Wang et al., 2024). Second, the cross-sectional design of the present study restricts our ability to capture the dynamic trajectory of resilience over time. Prospective longitudinal studies would not only allow for a better understanding of how resilience evolves but also provide stronger evidence regarding potential causal relationships between resilience and neural processes. Third, the focus on young adults in our study may restrict the generalizability of the findings to broader populations. To overcome this limitation, future research should consider including more diverse samples, encompassing individuals from different age groups, such as clinical and older adult samples. Fourth, the divergent findings between the neutral and negative movies warrant cautious interpretation. Although the two movies differed significantly in emotional ratings, it remains unclear whether the observed specificity truly reflects sensitivity to negative content. Other movie-related factors—such as cognitive complexity—may also introduce variance that substantially shapes neural responses (J. Chen & Bornstein, 2024; X. Wang et al., 2025). Therefore, any conclusions that appear specific to the negative movie should be considered preliminary and require replication in future studies that systematically control or counterbalance additional stimulus properties, such as narrative coherence and perceptual load. Lastly, although negative movies reliably evoke emotional responses, they primarily capture participants’ immediate reactions and do not assess post-event or regulatory processes—core components of resilience. Future studies would benefit from incorporating paradigms that measure both initial responses and subsequent recovery, or from employing designs that more effectively isolate resilience-related subprocesses.

In conclusion, the current study revealed that resilience plays a role in eliciting neural similarity among individuals during movie viewing. This resilience-related neural similarity aligns with the AnnaK principle, wherein resilient individuals share similar neural responses, in a synchronized manner with coherence among multiple brain networks involving attention, cognitive control, and social cognition, while low-resilience individuals exhibit divergent neural activity and lower similarity among themselves. Moreover, we propose the effect of IU on resilience-related neural similarity, which highlights the role of IU in resilience-related processes and its associated brain responses. This knowledge deepens our understanding of resilience and may potentially inform clinical and therapeutic interventions aimed at individualized approaches to fostering resilience.

## Supplementary Material

Supplementary Material

## Data Availability

Behavioral scores, ROI time series, and analysis codes are available at OSF | Video watching and resilience (https://osf.io/cgjkf/). Original MRI data will be available upon request from the senior corresponding author M.Z. as approval from the local ethics committee is required for access to the original data. The outline of proposal to access original MRI data should be specified for such approval.
