# Supplementary material for: Resilience-related neural similarity during naturalistic movie watching
Source: Imaging Neurosci (Camb). 2026 Mar 16;4:IMAG.a.1165. doi: 10.1162/IMAG.a.1165 (PMC12994002; doi:10.1162/IMAG.a.1165)
Supplement: Supplementary Material [file IMAG.a.1165_supp.pdf]

Supplementary

Resilience-related neural similarity during naturalistic movie watching

Shuer Ye, Leona Rahel Bätz, Avneesh Jain, Alireza Salami, Maryam Ziaei

* Corresponding author: Shuer Ye, Maryam Ziaei
Email: shuer.ye@ntnu.no, maryam.ziaei@ntnu.no

This file includes:

Supplementary information

Figure S1-S4

Table S1-S10

References

Supplementary information

Pilot experiment for selecting movie clips

Four movie clips, each lasting about 8 minutes, were sourced from the internet and previous studies (**Tab.S1**, Taylor et al., 2017). The first clip was a documentary showcasing various animals accompanied by soft background music entitled "*Animals*". The second clip, titled "*Pottery"*, documents the processing of two women making pottery. The third clip is a story of a woman desperately attempting to maintain her balance on a slope of a dam to prevent herself from plummeting into the abyss below entitled "*Curve*". The fourth is a clip from the famous black-white movie "*Bang! You're Dead"* from 1961*(directed by Alfred Hitchcock)*, tells a thrilling story that A 5-year-old boy finds his uncle's revolver, partially loads it with bullets, and plays with it in public, unaware of its deadly power. Based on the content and previous studies, the movie *Animals* and *Pottery* are considered as neutral clips, while *Curve* and *Bang! You're* dead are considered as negative ones.

To examine the emotional property, including the emotional valence and arousal of the movie clips, a pilot experiment was conducted. We recruited 24 participants (12 males, mean age =25.5 ±3.96 yrs) from the local community to rate these four movies. A MATLAB-based software for Continuous Affect Rating and Media Annotation (CARMA, v14.10) was used to play the movies and to acquire continuous ratings on emotional intensity ranging from 0 to 100 (Girard, 2014). During the movie watching, participants were asked to indicate their emotional intensity they experienced by adjusting a cursor on a scroll in the bottom of the screen. The rating feedback was sampled at the rate of 1 Hz (i.e., sample the position of the cursor one time per second). Following watching each movie, they provided ratings on the intensity of various emotions (e.g., happiness, anger, stress, satisfaction, etc.) as well as their perception of emotional valence and arousal induced by the movies.

To avoid the spillover effect, the neutral movie clips were presented first, followed by the negative movies. The sequence of movie clips within the same condition was counterbalanced across participants (see **Fig.S1a**). None of the participants had watched any of these movie clips before the experiment.

The results of continuous emotion-intensity feedback are presented in **Fig.S1b&c.** For the neutral movie clips, *Pottery* triggered lower emotional intensity than *Animals* (t = -16.304, p < 0.001, d = 3.033). For negative movies, participants experienced higher emotion intensity in *Curve* rather than *Bang! You're dead* (t = 45.377*,* p < 0.001, d = 2.911). In the current sample, the average-rating consistency intraclass correlation coefficient is 0.823 (*Animals*), 0.818 (*Pottery*),0.976 (*Curve*), and 0.969 (*Bang! You're Dead*), indicating good reliability of the continuous rating for all movie clips.

The results of the emotion rating following movie watching are presented in **Fig.S1d&e**. Participants predominantly reported feeling happy, interested, relaxed, and satisfied while watching both neutral movies. The overall intensity of these major emotions in *Pottery* is lower than in *Animals* indicating *Animals* induced stronger emotion experience. In the comparison between two neutral movies, *Animals* and *Pottery*, significantly higher emotional valence

($mean_{Animals}$ = 6.917, $mean_{Pottery}$ = 5.875, t = 3.824, p < 0.001, d =0.781) and arousal ($mean_{Animals}$ = 4.083, $mean_{Pottery}$ = 2.958, t = 2.944, p = 0.007, d = 0.601) were observed in *Animals* compared to *Pottery.* In the negative movie clips, participants reported high level of stress, fear, and pain during the *Curve*, while *Bang! You're Dead* elicited feelings of stress, fear, and anger in participants. Moreover, participants reported overall higher valence and lower arousal for the negative movies compared to neutral ones. In the comparison between two negative movies, *Curve* and *Bang! You're Dead,* a marginally significant difference was observed in emotional valence ($mean_{Curve}$ = 3.125, $mean_{Bang!\ You're\ Dead}$ = 3.750, t = -2.044, p = 0.051, d = 0.417) while no differences in arousal ($mean_{Curve}$ = 6.542, $mean_{Bang!\ You're\ Dead}$ = 6.125, t = 1.551, p = 0.134, d =0.317) were obtained. Taken together, the movies we considered as neutral (i.e., *Animals* and *Pottery*) were distinctively different from the negative movie clips (i.e., *Curve* and *Bang!You're Dead*). Compared with negative movies, neutral movies induced fewer negative emotions, higher emotional valence, lower emotional arousal, and lower continuous experienced emotional intensity during the viewing.

We further investigated sex differences in the ratings of these clips (**Tab.S10**). Across the four clips, most sex differences in emotional valence and continuous intensity ratings were small and not statistically significant. However, several notable differences emerged for arousal ratings. Female participants reported significantly higher arousal than males for *Animals* and *Curve* and showed a markedly higher arousal rating for *Bang! You're Dead*. For valence, a significant sex difference was observed only for *Bang! You're Dead*, where males rated the clip as more positive. No significant sex differences were found in continuous intensity ratings for any clip.

Most previous fMRI experiments used nature documentaries similar to *Animals*, as a neutral condition(Camacho et al., 2019; Chen et al., 2020; van Baar et al., 2021). In our experiment, however, we opted to use *Pottery* as the neutral stimulus for the following reasons: 1) Based on the results of the emotion rating, *Animals* received more positive ratings on emotional valence and evoked greater emotional arousal and intensity in the audience compared with *Pottery*, suggesting that it leans more towards being a positive stimulus instead of a neutral one.2) In *Animals*, soft background music accompanies the scenes, whereas in *Pottery*, only the real sounds from the scenes, such as the characters' actions, are present without any additional music making this clip similar to negative stimuli and closer to real life. 3) Finally, to have more comparison with the negative movie clips which included human faces, the Animals did not include any human faces and thus the pottery was chosen instead.

For negative movies, although *Bang! You're Dead* has been applied as the negative movie to induce tension and stress in previous studies (Taylor et al., 2017), we used *Curve* rather than *Bang! You're Dead* with the following reasons: 1). Based on the results, *Curve* triggered stronger and more types of negative emotions than *Bang! You're Dead*, 2) *Bang! You're Dead is* predominantly spoken in English, and this language preference may impact the information processing of non-native English speakers. On the other hand, *Curve* is a movie without dialogue, making it more accessible and compatible to speakers of other languages.

Moreover, the selected movies (i.e., *Pottery* and *Curve*) shared the following characteristics to control for potential confounding effects: 1) No more than one human character is present in each

clip at any given time; 2) all the depicted characters are females; 3) Absence of dialogue, word descriptions, and subtitles. 4) Both movies are presented in color.

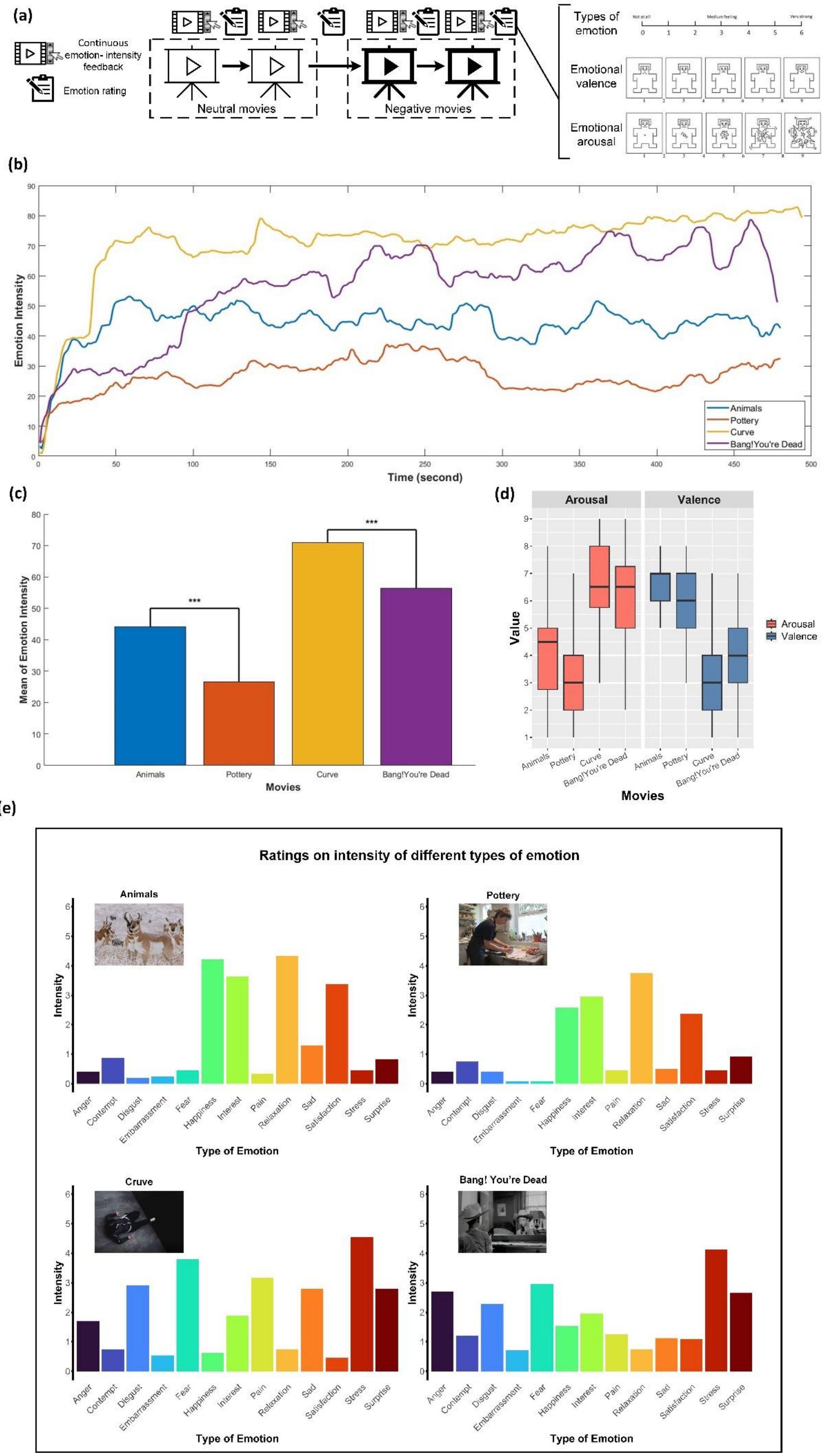
(a)
Continuous emotion- intensity feedback
Emotion rating
Neutral movies
Negative movies
Types of emotion
Not at all
Medium feeling
Very strong
0 1 2 3 4 5 6
Emotional valence
Emotional arousal
(b)
Emotion Intensity
Time (second)
Animals
Pottery
Curve
Bang!You're Dead
(c)
Mean of Emotion Intensity
***
***
Animals
Pottery
Curve
Bang!You're Dead
Movies
(d)
Arousal
Valence
Value
Arousal
Valence
Animals
Pottery
Curve
Bang!You're Dead
Movies
(e)
Ratings on intensity of different types of emotion
Animals
Pottery
Cruve
Bang! You're Dead
Intensity
Anger
Contempt
Disgust
Embarrassment
Fear
Happiness
Interest
Pain
Relaxation
Sad
Satisfaction
Stress
Surprise
Type of Emotion

Figure S1. Workflow and results of the movie-rating. **(a)** Participants were instructed to watch 4 movie clips (2 neutral and 2 negative movies). During the viewing process, participants provided continuous feedback regarding the emotional intensity they experienced. After watching each movie, they rated the intensity of various emotions, as well as emotional valence and arousal. **(b)** Changes in emotion intensity induced by the movies over the course of movie clips indicating lower intensity overall for the *Pottery* and highest for the *Curve*. Panel **(c)** represents the bar plot of the mean of the emotion intensity for each movie, indicating higher emotional intensity for the *Curve* and *Animals* compared to the *Bang! You're dead* and *Pottery*, respectively. Panel **(d)** represents the results of ratings on emotional valence and arousal. The lower and upper edges of the box plot represent the first quartile and third quartile, respectively. The line inside the box represents the median. Panel **(e)** indicates the results of ratings on various emotions experienced during movie-watching. ***: $p < 0.001$

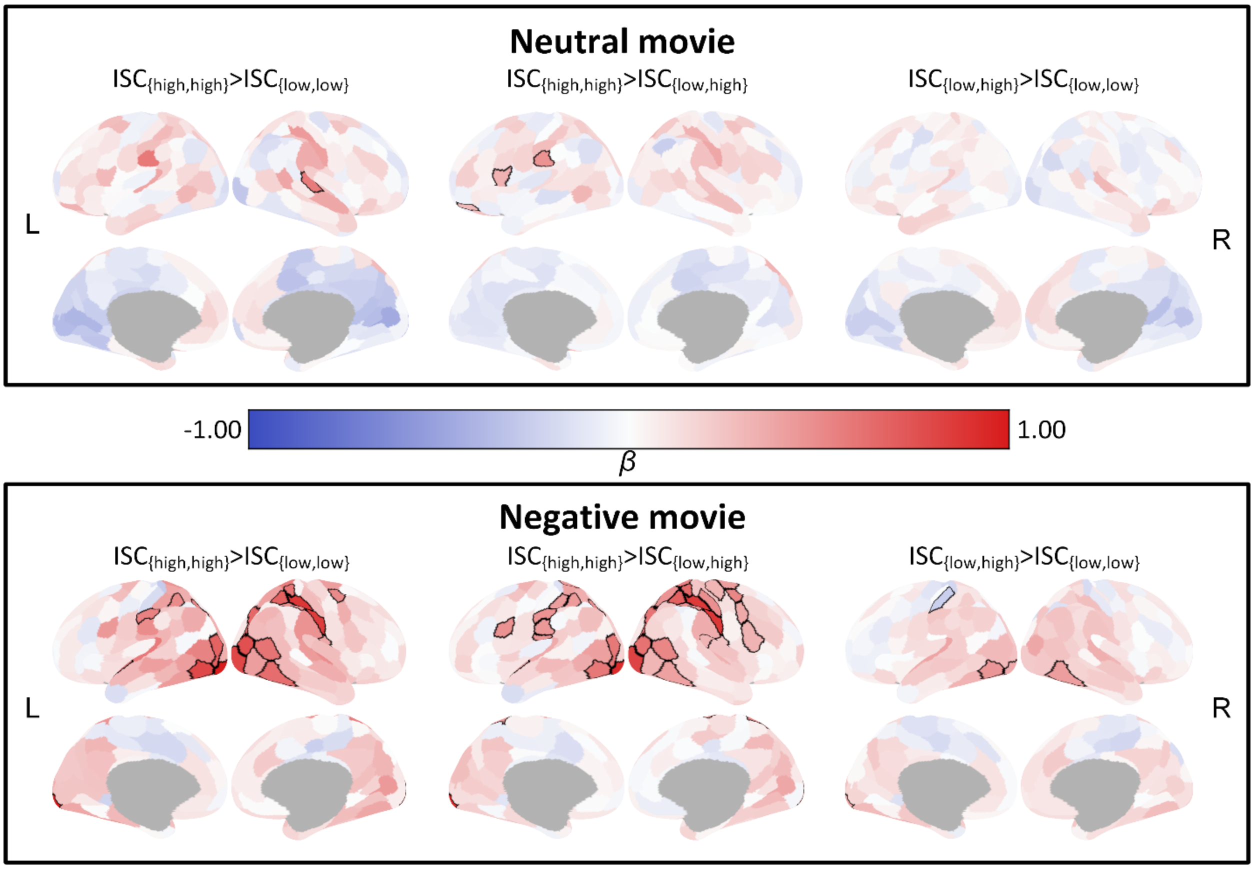

Figure S2. Dyad-level analysis of ISC with binarized resilience score (N=32). Resilience scores were binarized according to the lower and upper quartiles (i.e., Participants with scores above 80 were classified into the high resilience group, whereas those scoring 60 or below were classified into the low resilience group. Thirty participants who scored between 60 and 80 were excluded from this validation analysis. The β-value reflects the estimated mean difference in ISC between the dyad types specified in each contrast. Regions showing significant differences between the two dyad types after false discovery rate (FDR) correction are outlined in black.

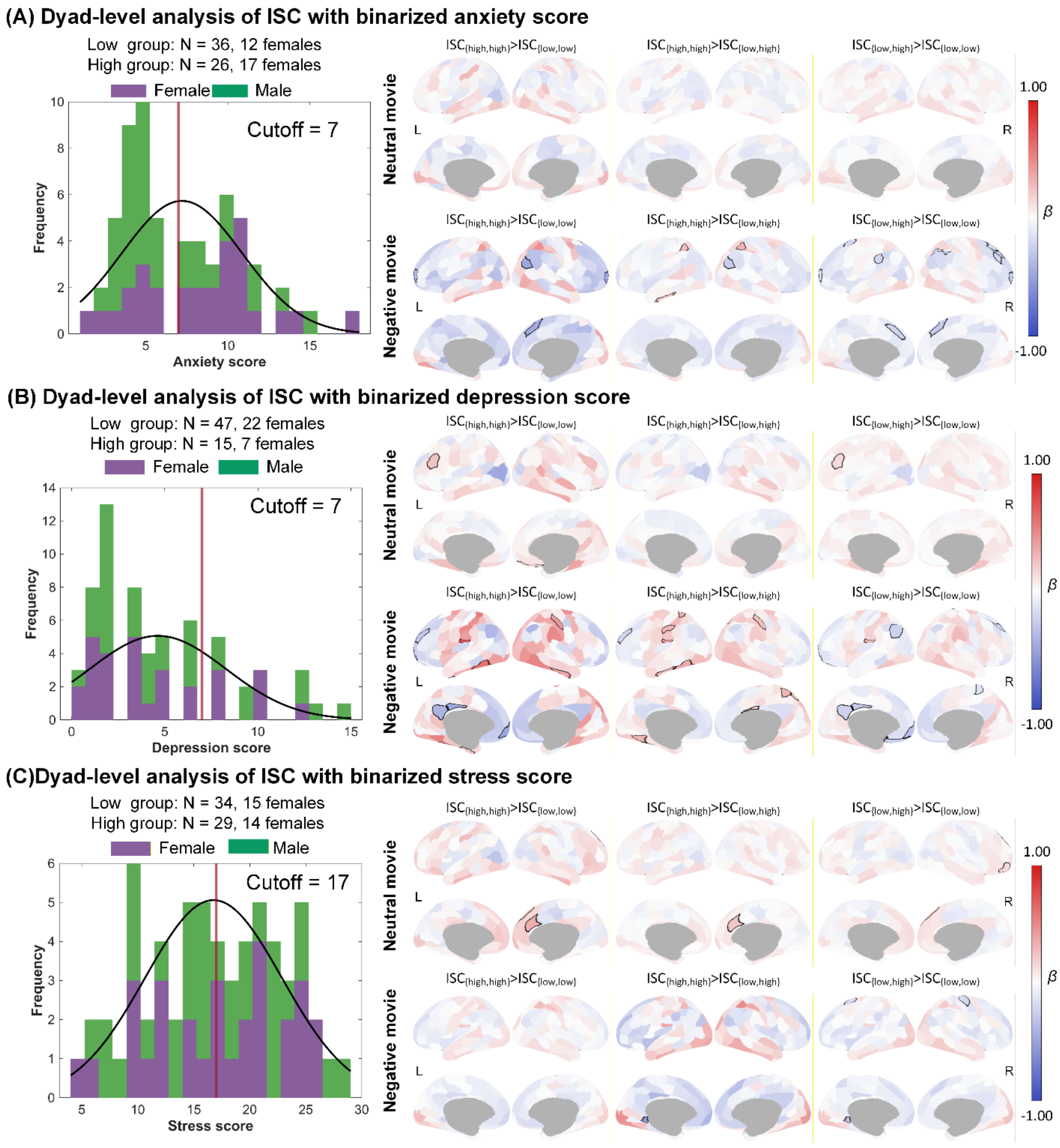

Figure S3. Dyad-level analysis of ISC with binarized anxiety (A), depression (B), and stress (C) score. Anxiety and depression scores, measured by the Hospital Anxiety and Depression Scale, were binarized according to the clinical cut-off of 7 (i.e., Participants with scores above 7 were classified into the high group, indicating the presence of anxiety/depressive symptoms, whereas those scoring 7 or below were classified into the low group, indicating the absence of such symptoms), whereas stress scores, measured by Perceived Stress Scale, were binarized based on a median (M=17) split. The β-value reflects the estimated mean difference in ISC between the dyad types specified in each contrast. Regions showing significant differences between the two dyad types after false discovery rate (FDR) correction are outlined in black.

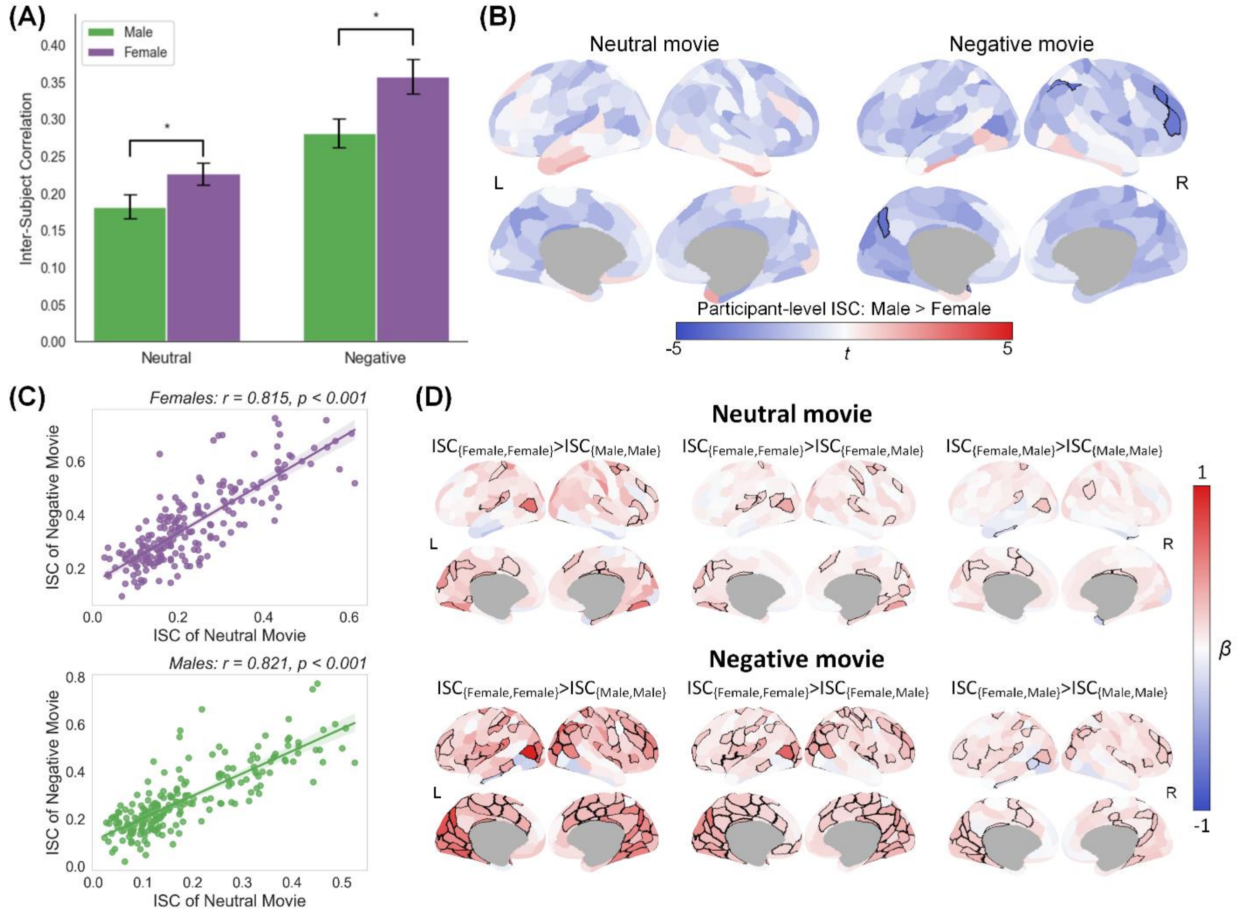

Figure S4. Sex effects on inter-subject correlation. **(A)** The sex differences in global participant-level ISC. The asterisk indicates statistical significance at $p < 0.05$. **(B)** The sex differences in participant-level ISC at regional level. Regions showing significant sex differences after false discovery rate (FDR) correction are outlined in black. **(C)** The correlation between the ISC maps of the two movie conditions within each sex group. **(D)** The results of the IS-RSA and the planned contrasts comparing ISC across the three dyad types: {Female, Female}, {Female, Male}, {Male, Male}. The β-value reflects the estimated mean difference in ISC between the dyad types specified in each contrast. Regions showing significant differences between the two dyad types after FDR correction are outlined in black.

Table S1. The website for each movie clips.

Movie clips	Website
Animals	https://www.youtube.com/watch?v=7IBzc3pajzY&list=PLJtPH8t_isfXtrfesaF0n5CnneIqPfSTt&index=15
Pottery	https://www.youtube.com/watch?v=Qi4JQEBW6Kw
Curve	https://www.youtube.com/watch?v=2dD3Fawk4y0
Bang!You're Dead	-

Table S2. Participant-level ISC of subcortical areas.

Subcortical region	ISC (Mean ± SD)		*t*-value
	Neutral movie	Negative movie	
Hippocampus (R)	0.126 ± 0.159	0.257 ± 0.192	-4.826*
Amygdala (R)	0.155 ± 0.138	0.283 ± 0.151	-6.248*
Posterior Thalamus (R)	0.179 ± 0.162	0.269 ± 0.186	-3.894
Anterior Thalamus (R)	0.208 ± 0.141	0.274 ± 0.152	-3.086
Nucleus accumbens (R)	0.151 ± 0.139	0.173 ± 0.160	-0.915
Globus pallidus (R)	0.071 ± 0.113	0.213 ± 0.218	-4.848*
Putamen (R)	0.036 ± 0.151	0.211 ± 0.186	-5.626*
Caudate nucleus (R)	0.058 ± 0.127	0.218 ± 0.202	-5.752*
Hippocampus (L)	0.148 ± 0.186	0.239 ± 0.197	-3.402
Amygdala (L)	0.141 ± 0.127	0.255 ± 0.154	-5.295*
Posterior Thalamus (L)	0.150 ± 0.178	0.264 ± 0.191	-4.167
Anterior Thalamus (L)	0.179 ± 0.137	0.251 ± 0.168	-3.402
Nucleus accumbens (L)	0.167 ± 0.142	0.179 ± 0.156	-0.619
Globus pallidus (L)	0.059 ± 0.116	0.208 ± 0.203	-5.265*
Putamen (L)	0.032 ± 0.135	0.200 ± 0.178	-5.815*
Caudate nucleus (L)	0.082 ± 0.129	0.230 ± 0.183	-5.757*

FDR correction was applied to correct for multiple comparisons. All the reported *p*-values are two-tailed.

SD: standard deviation; R: right; L: left; *: FDR-corrected $p < 0.05$.

Table S3. Dyad-level results that relate binarized resilience with ISC during neutral movie.

Region	Estimated mean difference	Standard Error	Corrected p-value	Network
**Contrast: $ISC_{\{high, high\}} > ISC_{\{low, low\}}$**				
Significant cortical regions				
No. 93 PFCv_2	0.231	0.085	0.025	DMN
No. 17 SomMotA_5	0.319	0.124	0.028	SMN
No. 139 PostC_3	0.455	0.180	0.028	DAN
No. 39 FEF_1	0.439	0.184	0.044	DAN
Subcortical regions				
Hippocampus (R)	0.071	0.111	0.609	SUB
Amygdala (R)	-0.035	0.106	0.823	SUB
Posterior Thalamus (R)	0.000	0.128	0.997	SUB
Anterior Thalamus (R)	0.146	0.124	0.322	SUB
Nucleus accumbens (R)	-0.106	0.105	0.387	SUB
Globus pallidus (R)	0.032	0.066	0.706	SUB
Putamen (R)	0.010	0.079	0.938	SUB
Caudate nucleus (R)	-0.055	0.073	0.545	SUB
Hippocampus (L)	0.024	0.135	0.906	SUB
Amygdala (L)	0.002	0.098	0.992	SUB
Posterior Thalamus (L)	0.039	0.125	0.832	SUB
Anterior Thalamus (L)	0.121	0.109	0.342	SUB
Nucleus accumbens (L)	-0.078	0.116	0.602	SUB
Globus pallidus (L)	-0.008	0.066	0.939	SUB
Putamen (L)	0.032	0.069	0.720	SUB
Caudate nucleus (L)	-0.057	0.079	0.567	SUB
**Contrast: $ISC_{\{high, high\}} > ISC_{\{low, high\}}$**				
Significant cortical regions				
No. 140 PostC_4	0.362	0.118	0.010	DAN
No. 153 PFCl_1	0.190	0.067	0.019	CN

No. 17 SomMotA_5	0.206	0.077	0.025	SMN
No. 38 PostC_4	0.290	0.114	0.028	DAN
No. 114 SomMotA_2	0.178	0.070	0.028	SMN
No. 139 PostC_3	0.249	0.099	0.028	DAN
No. 141 FEF_1	0.277	0.111	0.028	DAN
Subcortical regions				
Hippocampus (R)	-0.030	0.072	0.759	SUB
Amygdala (R)	-0.042	0.071	0.627	SUB
Posterior Thalamus (R)	-0.013	0.078	0.913	SUB
Anterior Thalamus (R)	0.115	0.077	0.206	SUB
Nucleus accumbens (R)	-0.027	0.070	0.777	SUB
Globus pallidus (R)	0.067	0.058	0.326	SUB
Putamen (R)	0.036	0.061	0.645	SUB
Caudate nucleus (R)	-0.006	0.060	0.948	SUB
Hippocampus (L)	-0.053	0.081	0.607	SUB
Amygdala (L)	-0.042	0.068	0.613	SUB
Posterior Thalamus (L)	-0.008	0.077	0.941	SUB
Anterior Thalamus (L)	0.078	0.071	0.349	SUB
Nucleus accumbens (L)	-0.060	0.074	0.501	SUB
Globus pallidus (L)	-0.007	0.058	0.939	SUB
Putamen (L)	0.038	0.059	0.607	SUB
Caudate nucleus (L)	-0.039	0.062	0.609	SUB
**Contrast: $ISC_{\{low, high\}}$ > $ISC_{\{low, low\}}$**				
Subcortical regions				
Hippocampus (R)	0.101	0.072	0.214	SUB
Amygdala (R)	0.008	0.071	0.939	SUB
Posterior Thalamus (R)	0.014	0.078	0.906	SUB
Anterior Thalamus (R)	0.031	0.077	0.763	SUB
Nucleus accumbens (R)	-0.079	0.070	0.326	SUB
Globus pallidus (R)	-0.035	0.058	0.609	SUB

Putamen (R)	-0.025	0.061	0.752	SUB
Caudate nucleus (R)	-0.049	0.060	0.480	SUB
Hippocampus (L)	0.077	0.081	0.411	SUB
Amygdala (L)	0.045	0.068	0.601	SUB
Posterior Thalamus (L)	0.047	0.077	0.613	SUB
Anterior Thalamus (L)	0.043	0.071	0.617	SUB
Nucleus accumbens (L)	-0.017	0.074	0.872	SUB
Globus pallidus (L)	-0.001	0.058	0.993	SUB
Putamen (L)	-0.006	0.059	0.939	SUB
Caudate nucleus (L)	-0.018	0.062	0.836	SUB

The index of cortical regions is taken from Schafer 200 atlas. FDR correction was applied to correct for multiple comparisons. All the reported *p*-values are two-tailed. R: right; L: left; DAN: dorsal attention network; CN: control network; DMN: default mode network; SMN: somatomotor network; SUB: subcortical network.

Table S4. Dyad-level results that relate binarized resilience with ISC during negative movie.

Region	Estimated mean difference	SE	Corrected *p*-value	Network
**Contrast: $ISC_{\{high, high\}} > ISC_{\{low, low\}}$**				
Significant cortical region				
No. 135 SPL_3	0.708	0.235	0.001	DAN
No. 138 PostC_2	0.848	0.290	0.002	DAN
No. 2 ExStr_2	0.728	0.254	0.002	VN
No. 137 Post_C1	0.771	0.275	0.003	DAN
No. 140 Post_C4	0.709	0.263	0.006	DAN
No. 117 SomMotA_5	0.725	0.272	0.006	SMN
No. 104 ExStr_2	0.618	0.245	0.011	VN
No. 79 pCunPCC_3	0.434	0.184	0.019	DMN
No. 64 PFCl_2	0.498	0.215	0.020	CN
No. 68 IPL_1	0.408	0.177	0.021	CN
No. 182 pCun_2	0.448	0.196	0.021	CN
No. 181 pCun_1	0.509	0.226	0.024	CN
No. 36 PostC_2	0.588	0.263	0.024	DAN
No. 118 SomMotA_6	0.252	0.113	0.024	SMN
No. 184 IPL_1	0.448	0.202	0.025	DMN
No. 60 IPS_3	0.487	0.224	0.027	CN
No. 166 IPS_2	0.471	0.217	0.028	CN
No. 172 Temp_2	0.399	0.187	0.029	CN
No. 31 ParOcc_1	0.609	0.289	0.033	DAN
No. 75 IPL_1	0.386	0.186	0.035	DMN
No. 77 pCunPCC_1	0.349	0.168	0.035	DMN
No. 142 ParOper_1	0.550	0.268	0.035	VAN
No. 72 pCun_1	0.430	0.211	0.035	CN
No. 115 SomMotA_3	0.320	0.157	0.035	SMN

No. 5 ExStr_4	0.532	0.263	0.036	VN
No. 16 SomMotA_4	0.279	0.139	0.037	SMN
No. 186 pCunPCC_1	0.384	0.192	0.038	DMN
No. 39 EFE_1	0.336	0.170	0.041	DAN
No. 37 PostC_3	0.565	0.289	0.043	DAN
No. 67 Temp_1	0.334	0.171	0.043	CN
No. 120 SomMotA_8	0.491	0.254	0.046	SMN
No. 113 SomMotA_1	0.332	0.173	0.047	SMN
No. 86 Temp_4	0.402	0.201	0.047	DMN
No. 173 IPL_1	0.400	0.210	0.048	CN
Subcortical region				
Hippocampus (R)	0.236	0.192	0.228	SUB
Amygdala (R)	0.060	0.164	0.753	SUB
Posterior Thalamus (R)	-0.128	0.189	0.520	SUB
Anterior Thalamus (R)	-0.066	0.163	0.722	SUB
Nucleus accumbens (R)	-0.021	0.137	0.902	SUB
Globus pallidus (R)	0.103	0.189	0.621	SUB
Putamen (R)	-0.199	0.170	0.252	SUB
Caudate nucleus (R)	-0.087	0.182	0.671	SUB
Hippocampus (L)	0.154	0.188	0.421	SUB
Amygdala (L)	0.201	0.150	0.189	SUB
Posterior Thalamus (L)	-0.076	0.190	0.726	SUB
Anterior Thalamus (L)	-0.030	0.165	0.889	SUB
Nucleus accumbens (L)	-0.194	0.125	0.119	SUB
Globus pallidus (L)	-0.050	0.184	0.823	SUB
Putamen (L)	-0.161	0.157	0.320	SUB
Caudate nucleus (L)	-0.163	0.172	0.344	SUB
**Contrast: $ISC_{\{high, high\}} > ISC_{\{low, high\}}$**				
Significant cortical region				
No. 135 SPL_3	0.476	0.123	<0.001	DAN

No. 2 ExStr_2	0.448	0.131	<0.001	VN
No. 138 PostC_2	0.506	0.147	<0.001	DAN
No. 182 pCun_2	0.371	0.106	<0.001	CN
No. 104 ExStr_3	0.422	0.127	<0.001	VN
No. 137 PostC_1	0.459	0.140	<0.001	DAN
No. 140 PostC_4	0.432	0.135	0.001	DAN
No. 117 SomMotA_5	0.441	0.139	0.001	SMN
No. 79 pCunPCC_3	0.315	0.101	0.001	DMN
No. 64 PFCl_2	0.335	0.114	0.002	CN
No. 165 IPS_1	0.272	0.094	0.002	CN
No. 166 IPS_2	0.314	0.115	0.005	CN
No. 86 Temp_4	0.292	0.112	0.008	DMN
No. 115 SomMotA_3	0.233	0.090	0.008	SMN
No. 36 PostC_2	0.349	0.135	0.009	DAN
No. 75 IPL_1	0.256	0.102	0.011	CN
No. 181 pCun_1	0.298	0.119	0.011	CN
No. 12 ExStrSup_2	0.301	0.124	0.016	CN
No. 68 IPL_1	0.237	0.098	0.016	CN
No. 118 SomMotA_6	0.175	0.073	0.016	SMN
No. 60 IPS_3	0.280	0.118	0.019	CN
No. 32 SPL_1	0.276	0.117	0.019	DAN
No. 58 IPS_1	0.180	0.077	0.020	CN
No. 173 IPL_1	0.261	0.112	0.020	CN
No. 5 ExStr_4	0.309	0.135	0.021	VN
No. 31 ParOcc_1	0.335	0.147	0.021	DAN
No. 67 Temp_1	0.217	0.096	0.021	CN
No. 172 Temp_2	0.234	0.102	0.021	CN
No. 175 PFCld_1	0.186	0.082	0.021	CN
No. 16 SomMotA_4	0.183	0.082	0.024	SMN
No. 133 SPL_1	0.313	0.142	0.025	DAN

No. 109 StriCal_1	0.289	0.132	0.026	VN
No. 120 SomMotA_8	0.287	0.131	0.027	SMN
No. 113 SomMotA_1	0.208	0.096	0.027	SMN
No. 111 ExStrSup_2	0.272	0.126	0.028	VN
No. 37 PostC_3	0.313	0.147	0.031	DAN
No. 39 FEF_1	0.195	0.095	0.035	DAN
No. 42 FrOper_1	0.204	0.099	0.035	VAN
No. 59 IPS_2	0.184	0.090	0.035	CN
No. 72 pCun_1	0.230	0.112	0.035	CN
No. 77 pCunPCC_1	0.194	0.094	0.035	DMN
No. 136 SPL_4	0.273	0.133	0.035	DAN
No. 142 ParOper_1	0.283	0.138	0.035	VAN
No. 184 IPL_1	0.224	0.109	0.035	DMN
No. 176 PFCld_2	0.189	0.093	0.037	CN
No. 143 PrC_1	0.211	0.105	0.038	VAN
No. 26 S2_3	0.237	0.121	0.043	SMN
No. 195 Rsp_1	0.255	0.130	0.043	DMN
No. 38 PostC_4	0.244	0.127	0.047	DAN
No. 40 ParOper_1	0.255	0.133	0.047	VAN
No. 186 pCunPCC_1	0.200	0.104	0.047	DMN
Subcortical region				
Hippocampus (R)	0.121	0.104	0.253	SUB
Amygdala (R)	0.028	0.093	0.797	SUB
Posterior Thalamus (R)	-0.057	0.103	0.621	SUB
Anterior Thalamus (R)	-0.044	0.092	0.671	SUB
Nucleus accumbens (R)	-0.118	0.082	0.147	SUB
Globus pallidus (R)	0.021	0.103	0.878	SUB
Putamen (R)	-0.088	0.095	0.364	SUB
Caudate nucleus (R)	-0.083	0.100	0.418	SUB
Hippocampus (L)	0.036	0.103	0.762	SUB

Amygdala (L)	0.096	0.087	0.280	SUB
Posterior Thalamus (L)	-0.089	0.103	0.399	SUB
Anterior Thalamus (L)	-0.038	0.093	0.719	SUB
Nucleus accumbens (L)	-0.114	0.077	0.136	SUB
Globus pallidus (L)	0.006	0.101	0.958	SUB
Putamen (L)	-0.094	0.090	0.308	SUB
Caudate nucleus (L)	-0.094	0.096	0.337	SUB
**Contrast: $ISC_{\{low, high\}}$ > $ISC_{\{low, low\}}$**				
Significant cortical region				
No. 138 PostC_2	0.341	0.147	0.020	DAN
No. 137 PostC_1	0.312	0.140	0.024	DAN
No. 2 ExStr_2	0.280	0.131	0.029	VN
No. 29 TempOcc_1	0.250	0.121	0.035	DAN
No.117 SomMotA_5	0.284	0.139	0.035	SMN
No. 140 PostC_4	0.276	0.135	0.035	DAN
No. 184 IPL_1	0.224	0.109	0.035	DMN
No. 63 PFCl_1	-0.163	0.082	0.035	CN
No. 142 ParOper_1	0.267	0.138	0.043	VAN
No. 135 SPL_3	0.232	0.123	0.049	DAN
Subcortical region				
Hippocampus (R)	0.114	0.104	0.276	SUB
Amygdala (R)	0.032	0.093	0.762	SUB
Posterior Thalamus (R)	-0.072	0.103	0.507	SUB
Anterior Thalamus (R)	-0.022	0.092	0.844	SUB
Nucleus accumbens (R)	0.097	0.082	0.231	SUB
Globus pallidus (R)	0.082	0.103	0.427	SUB
Putamen (R)	-0.111	0.095	0.245	SUB
Caudate nucleus (R)	-0.004	0.100	0.969	SUB
Hippocampus (L)	0.118	0.103	0.252	SUB
Amygdala (L)	0.105	0.087	0.229	SUB

Posterior Thalamus (L)	0.013	0.103	0.913	SUB
Anterior Thalamus (L)	0.008	0.093	0.942	SUB
Nucleus accumbens (L)	-0.080	0.077	0.300	SUB
Globus pallidus (L)	-0.056	0.101	0.611	SUB
Putamen (L)	-0.066	0.090	0.469	SUB
Caudate nucleus (L)	-0.069	0.096	0.483	SUB

The index of cortical regions is taken from Schafer 200 atlas. FDR correction was applied to correct for multiple comparisons. All the reported *p*-values are two-tailed. R: right; L: left; DAN: dorsal attention network; CN: control network; DMN: default mode network; SMN: somatomotor network; SUB: subcortical network; VN: visual network. VAN: ventral attention network.

Table S5. Dyad-level results that relate resilience similarity with ISC during neutral movie.

Region	β	SE	Corrected *p*-value	Network
Significant cortical regions				
No. 34 SPL_3	0.179	0.040	0.002	DAN
No. 141 FEF_1	0.171	0.039	0.002	DAN
No. 38 PostC_4	0.153	0.039	0.006	DAN
No. 40 ParOper_1	0.153	0.038	0.006	VAN
No. 43 FrOper_2	0.104	0.027	0.008	VAN
No. 137 PostC_1	0.149	0.039	0.008	DAN
No. 135 SPL_3	0.146	0.039	0.008	DAN
No. 127 S2_2	0.067	0.019	0.016	SMN
No. 28 Cent_2	0.088	0.025	0.016	SMN
No. 36 PostC_2	0.134	0.040	0.016	DAN
No. 134 SPL_2	0.134	0.039	0.016	DAN
No. 59 IPS_2	0.115	0.035	0.022	CN
No. 138 PostC_2	0.125	0.039	0.022	DAN
No. 14 SomMotA_2	0.064	0.020	0.023	SMN
No. 35 PostC_1	0.124	0.040	0.023	DAN
No. 45 FrMed_1	-0.062	0.019	0.023	VAN
No. 60 IPS_3	0.124	0.039	0.023	CN
No. 107 ExStrInf_1	0.121	0.038	0.023	VN
No. 143 PrC_1	0.112	0.034	0.023	VAN
No. 140 PostC_4	0.123	0.040	0.024	DAN
No. 166 IPS_2	0.115	0.039	0.032	CN
No. 32 SPL_1	0.111	0.039	0.047	DAN
No. 37 PostC_3	0.112	0.040	0.047	DAN
Subcortical regions				
Hippocampus (R)	0.038	0.026	0.454	SUB
Amygdala (R)	-0.009	0.025	0.975	SUB

Posterior Thalamus (R)	-0.029	0.029	0.727	SUB
Anterior Thalamus (R)	-0.035	0.029	0.621	SUB
Nucleus accumbens (R)	-0.066	0.024	0.059	SUB
Globus pallidus (R)	0.017	0.016	0.706	SUB
Putamen (R)	0.006	0.019	0.975	SUB
Caudate nucleus (R)	0.008	0.018	0.970	SUB
Hippocampus (L)	0.026	0.030	0.791	SUB
Amygdala (L)	-0.007	0.024	0.975	SUB
Posterior Thalamus (L)	-0.030	0.029	0.706	SUB
Anterior Thalamus (L)	-0.020	0.026	0.842	SUB
Nucleus accumbens (L)	-0.072	0.026	0.059	SUB
Globus pallidus (L)	0.005	0.016	0.975	SUB
Putamen (L)	0.025	0.017	0.454	SUB
Caudate nucleus (L)	-0.011	0.019	0.909	SUB

The index of cortical regions is taken from Schafer 200 atlas. FDR correction was applied to correct for multiple comparisons. All the reported *p*-values are two-tailed. R: right; L: left; DAN: dorsal attention network; VAN: ventral attention network; CN: control network; VN: visual network; SMN: somatomotor network; SUB: subcortical network; VAN: ventral attention network..

Table S6. Dyad-level results that related resilience similarity with ISC during negative movie.

Region	β	SE	Corrected *p*-value	Network
Significant cortical region				
No. 137 PostC_1	0.315	0.036	<0.001	DAN
No. 138 PostC_2	0.283	0.034	<0.001	DAN
No. 2 ExStr_2	0.260	0.038	<0.001	VN
No. 104 ExStr_3	0.263	0.039	<0.001	VN
No. 135 SPL_3	0.261	0.039	<0.001	DAN
No. 26 S2_3	0.249	0.039	<0.001	SMN
No. 129 S2_4	0.200	0.033	<0.001	SMN
No. 5 ExStr_4	0.209	0.039	<0.001	VN
No. 117 SomMotA_5	0.193	0.037	<0.001	SMN
No. 113 SomMotA_1	0.180	0.035	<0.001	SMN
No. 143 PrC_1	0.185	0.037	<0.001	VAN
No. 64 PFCl_2	0.191	0.039	<0.001	CN
No. 166 IPS_2	0.185	0.039	<0.001	CN
No. 36 PostC_2	0.176	0.038	<0.001	DAN
No. 141 FEF_1	0.180	0.039	<0.001	DAN
No. 38 PostC_4	0.179	0.040	<0.001	DAN
No. 24 S2_2	0.147	0.032	<0.001	SMN
No. 37 PostC_3	0.155	0.036	<0.001	DAN
No. 133 SPL_1	0.161	0.038	<0.001	DAN
No. 146 FrOper_1	0.147	0.033	<0.001	VAN
No. 39 FEF_1	0.150	0.035	<0.001	DAN
No. 67 Temp_1	0.148	0.035	<0.001	CN
No. 35 PostC_1	0.162	0.039	<0.001	DAN
No. 132 ParOcc_1	0.157	0.040	0.001	DAN
No. 140 PostC_4	0.151	0.038	0.001	DAN

No. 18 SomMotA_6	0.153	0.039	0.001	SMN
No. 177 PFClv_1	0.146	0.037	0.001	CN
No. 147 FrMed_1	0.129	0.033	0.001	VAN
No. 112 ExStrSup_3	0.154	0.040	0.001	VN
No. 43 FrOper_2	0.129	0.033	0.001	VAN
No. 127 S2_2	0.152	0.039	0.001	SMN
No. 168 PFCl_1	0.147	0.038	0.001	CN
No. 86 Temp_4	0.147	0.039	0.001	DMN
No. 42 FrOper_1	0.139	0.037	0.001	VAN
No. 136 SPL_4	0.145	0.039	0.002	DAN
No. 70 PFClv_1	0.122	0.033	0.002	CN
No. 12 ExStrSup_2	0.147	0.040	0.002	VN
No. 68 IPL_1	0.130	0.036	0.002	CN
No. 105 ExStr_4	0.131	0.037	0.002	VN
No. 40 ParOper_1	0.138	0.039	0.003	VAN
No. 165 IPS_1	0.127	0.036	0.003	CN
No. 178 PFClv_2	0.131	0.038	0.004	CN
No. 52 OFC_2	0.126	0.036	0.004	LN
No. 115 SomMotA_3	0.118	0.034	0.004	SMN
No. 111 ExStrSup_2	0.136	0.040	0.004	VN
No. 32 SPL_1	0.131	0.040	0.005	DAN
No. 87 IPL_1	0.124	0.038	0.007	DMN
No. 182 pCun_2	0.125	0.039	0.007	CN
No. 79 pCunPCC_3	0.121	0.038	0.007	DMN
No. 157 OFC_1	0.113	0.036	0.008	LN
No. 62 PFClv_1	0.080	0.026	0.012	CN
No. 181 pCun_1	0.117	0.040	0.014	CN
No. 31 ParOcc_1	0.105	0.036	0.014	DAN
No. 16 SomMotA_4	0.094	0.031	0.015	SMN
No. 120 SomMotA_8	0.115	0.040	0.015	SMN

No. 109 StriCal_1	0.112	0.039	0.019	VN
No. 57 Temp_1	0.112	0.040	0.019	CN
No. 74 Cingp_1	0.108	0.038	0.020	CN
No. 75 IPL_1	0.106	0.038	0.020	DMN
No. 152 PFClv_1	0.088	0.031	0.021	VAN
No. 25 Aud_3	0.108	0.040	0.023	SMN
No. 175 PFCld_1	0.084	0.031	0.027	CN
No. 150 FrMed_2	0.094	0.035	0.027	VAN
No. 60 IPS_3	0.107	0.040	0.027	CN
No. 107 ExStrInf_1	0.105	0.040	0.029	VN
No. 158 OFC_2	0.099	0.037	0.029	LN
No. 119 SomMotA_7	0.075	0.028	0.033	SMN
No. 142 ParOper_1	0.098	0.038	0.034	VAN
No. 59 IPS_2	0.089	0.034	0.034	CN
No. 69 PFCl_1	0.081	0.032	0.038	CN
No. 176 PFCld_2	0.087	0.035	0.045	CN
No. 95 PFCv_4	0.091	0.037	0.045	DMN
Subcortical region				
Hippocampus (R)	0.016	0.038	0.816	SUB
Amygdala (R)	0.002	0.035	0.978	SUB
Posterior Thalamus (R)	0.018	0.038	0.804	SUB
Anterior Thalamus (R)	0.004	0.035	0.971	SUB
Nucleus accumbens (R)	0.000	0.031	0.997	SUB
Globus pallidus (R)	0.016	0.038	0.816	SUB
Putamen (R)	-0.015	0.036	0.816	SUB
Caudate nucleus (R)	-0.026	0.037	0.663	SUB
Hippocampus (L)	-0.027	0.038	0.663	SUB
Amygdala (L)	0.044	0.033	0.338	SUB
Posterior Thalamus (L)	-0.018	0.038	0.804	SUB
Anterior Thalamus (L)	-0.030	0.035	0.583	SUB

Nucleus accumbens (L)	-0.036	0.029	0.385	SUB
Globus pallidus (L)	-0.004	0.037	0.971	SUB
Putamen (L)	-0.019	0.034	0.758	SUB
Caudate nucleus (L)	0.007	0.036	0.923	SUB

The index of cortical regions is taken from Schafer 200 atlas. FDR correction was applied to correct for multiple comparisons. All the reported *p*-values are two-tailed. R: right; L: left; DAN: dorsal attention network; CN: control network; DMN: default mode network; SMN: somatomotor network; SUB: subcortical network; VN: visual network. LN: limbic network; VAN: ventral attention network.

Table S7. The modulation effects of intolerance of uncertainty on resilience-driven neural synchrony in the neutral movie.

Region	Interaction	SE	Corrected *p*-value	Network
Significant cortical region				
No. 28 Cent_2	-0.096	0.018	0.001	SMN
No. 143 FrOper_2	-0.082	0.018	0.001	VAN
No. 37 PostC_3	-0.064	0.015	0.002	DAN
No. 140 PostC_4	-0.067	0.016	0.002	DAN
No. 150 FrMed_2	-0.074	0.018	0.002	VAN
No. 134 SPL_2	-0.069	0.017	0.002	DAN
No. 114 SomMotA_2	-0.070	0.018	0.003	DAN
No. 198 TempPar_2	-0.067	0.018	0.006	TempPar
No. 123 SomMotA_11	-0.066	0.018	0.006	SMN
No. 99 TempPar_1	-0.066	0.018	0.006	TempPar
No. 20 SomMotA_8	-0.063	0.018	0.009	SMN
No. 22 Aud_2	-0.062	0.018	0.009	SMN
No. 12 ExStrSup_2	-0.056	0.016	0.009	VN
No. 139 PostC_3	-0.060	0.018	0.009	DAN
No. 38 PostC_4	-0.055	0.017	0.013	DAN
No. 15 SomMotA_3	-0.059	0.018	0.015	SMN
No. 19 SomMotA_7	-0.057	0.018	0.019	SMN
No. 136 SPL_4	-0.051	0.016	0.020	DAN
No. 119 SomMotA_7	-0.056	0.018	0.024	SMN
No. 27 Cent_1	-0.054	0.018	0.024	SMN
No. 166 IPS_2	-0.052	0.017	0.024	CN
No. 40 ParOper_1	-0.051	0.017	0.029	DAN
No. 44 ParMed_1	-0.054	0.018	0.029	VAN
No. 35 PostC_1	-0.047	0.016	0.033	VAN
No. 17 SomMotA_5	-0.052	0.018	0.033	SMN

No. 137 PostC_1	-0.043	0.015	0.033	DAN
No. 199 TempPar_3	-0.053	0.018	0.033	TempPar
No. 49 Ins_1	-0.049	0.017	0.036	VAN
No. 141 FEF_1	-0.050	0.018	0.040	DAN
Subcortical region				
Hippocampus (R)	0.038	0.026	0.454	SUB
Amygdala (R)	-0.009	0.025	0.975	SUB
Posterior Thalamus (R)	-0.029	0.029	0.727	SUB
Anterior Thalamus (R)	-0.035	0.029	0.621	SUB
Nucleus accumbens (R)	-0.066	0.024	0.059	SUB
Globus pallidus (R)	0.017	0.016	0.706	SUB
Putamen (R)	0.006	0.019	0.975	SUB
Caudate nucleus (R)	0.008	0.018	0.970	SUB
Hippocampus (L)	0.026	0.030	0.791	SUB
Amygdala (L)	-0.007	0.024	0.975	SUB
Posterior Thalamus (L)	-0.030	0.029	0.706	SUB
Anterior Thalamus (L)	-0.020	0.026	0.842	SUB
Nucleus accumbens (L)	-0.072	0.026	0.059	SUB
Globus pallidus (L)	0.005	0.016	0.975	SUB
Putamen (L)	0.025	0.017	0.454	SUB
Caudate nucleus (L)	-0.011	0.019	0.909	SUB

The index of cortical regions is taken from Schafer 200 atlas. FDR correction was applied to correct for multiple comparisons. All the reported *p*-values are two-tailed. R: right; L: left; DAN: dorsal attention network; CN: control network; VN: visual network; SMN: somatomotor network; SUB: subcortical network; VAN: ventral attention network; TempPar: temporal parietal area.

Table S8. The modulation effects of intolerance of uncertainty on resilience-driven neural synchrony in the negative movie.

Region	Interaction	SE	Corrected *p*-value	Network
Significant cortical region				
No. 14 SomMotA_2	-0.092	0.018	<0.001	SMN
No. 35 PostC_1	-0.080	0.016	<0.001	DAN
No. 38 PostC_4	-0.079	0.016	<0.001	DAN
No. 36 PostC_2	-0.058	0.014	0.003	DAN
No. 139 PostC_3	-0.067	0.017	0.004	DAN
No. 74 Cingp_1	0.066	0.017	0.004	CN
No. 5 ExStr_4	-0.055	0.014	0.005	VN
No. 126 S2_1	0.066	0.018	0.006	SMN
No. 156 PFCmp_1	0.062	0.017	0.009	VAN
No. 50 PFCmp_1	0.060	0.017	0.012	VAN
No. 61 PFCd_1	-0.061	0.018	0.014	CN
No. 33 SPL_2	-0.057	0.017	0.014	DAN
No. 37 PostC_3	-0.042	0.013	0.014	DAN
No. 82 PFCm_3	0.059	0.018	0.014	DMN
No. 78 pCunPCC_2	0.057	0.017	0.015	DMN
No. 72 pCun_1	0.055	0.017	0.016	CN
No. 167 PFCd_1	-0.058	0.018	0.017	CN
No. 26 S2_3	-0.050	0.016	0.022	SMN
No. 92 PFCv_1	0.054	0.017	0.022	DMN
No. 166 IPS_2	-0.051	0.016	0.024	CN
No. 67 Temp_1	0.054	0.018	0.024	CN
No. 88 PFCd_1	0.054	0.018	0.024	DMN
No. 181 pCun_1	0.049	0.016	0.025	CN
No. 154 Ins_1	0.049	0.016	0.026	VAN
No. 140 PostC_4	-0.041	0.014	0.033	DAN

No. 68 IPL_1	0.049	0.018	0.041	CN
Subcortical region				
Hippocampus (R)	0.016	0.017	0.622	SUB
Amygdala (R)	0.001	0.018	0.995	SUB
Posterior Thalamus (R)	-0.017	0.018	0.608	SUB
Anterior Thalamus (R)	-0.013	0.018	0.696	SUB
Nucleus accumbens (R)	0.052	0.018	0.036	SUB
Globus pallidus (R)	0.041	0.018	0.102	SUB
Putamen (R)	0.007	0.018	0.836	SUB
Caudate nucleus (R)	0.016	0.018	0.622	SUB
Hippocampus (L)	0.031	0.018	0.246	SUB
Amygdala (L)	0.031	0.018	0.264	SUB
Posterior Thalamus (L)	0.011	0.018	0.731	SUB
Anterior Thalamus (L)	0.008	0.018	0.826	SUB
Nucleus accumbens (L)	0.029	0.018	0.302	SUB
Globus pallidus (L)	0.021	0.018	0.528	SUB
Putamen (L)	0.006	0.018	0.855	SUB
Caudate nucleus (L)	0.011	0.018	0.734	SUB

The index of cortical regions is taken from Schafer 200 atlas. FDR correction was applied to correct for multiple comparisons. All the reported *p*-values are two-tailed. R: right; L: left; DAN: dorsal attention network; CN: control network; DMN: default mode network; SMN: somatomotor network; SUB: subcortical network; VN: visual network; VAN: ventral attention network.

Table S9. Sex differences in emotional rating and continues intensity rating of the four clips in the pilot study.

		Mean ± SD		
		**Male (N=12)**	**Female (N=12)**	**t-value**
Animals				
	Valence	6.75 ±1.14	7.08 ± 0.51	-0.92
	Arousal	3.25 ±1.66	4.92 ± 1.51	-2.58*
	Intensity	42.39 ±19.24	43.27 ± 18.61	-0.11
Pottery				
	Valence	5.92 ±1.16	5.83 ± 1.27	0.17
	Arousal	3.00 ±1.65	2.92 ± 1.08	0.15
	Intensity	29.12 ±12.90	22.92 ± 14.44	1.09
Curve				
	Valence	3.50 ± 1.57	2.75 ± 1.54	1.18
	Arousal	5.67 ± 2.02	7.42 ± 1.24	-2.56*
	Intensity	64.11 ± 13.87	78.82 ± 21.86	-1.95
Bang!You're Dead				
	Valence	4.41 ± 1.44	3.08 ± 1.56	2.17*
	Arousal	4.91 ± 1.83	7.33 ± 0.89	-4.11***
	Intensity	50.47 ± 9.81	62.52 ± 18.32	-1.99

Note: SD = standard deviation. *: $p<0.05$; **: $p<0.01$; ***: $p<0.001$.

Table S10. Sex difference in behavior measures in the main study.

	Mean ± SD		t-value
	Male (N=33)	Female (N=29)	
**Emotional Valence**			
Baseline	6.06 ± 1.17	6.24 ± 1.53	-0.53
Neutral (*Pottery*)	6.24 ± 1.39	6.00 ± 1.17	0.74
Negative (*Curve*)	3.97 ± 1.31	3.69 ± 1.71	0.73
**Emotional Arousal**			
Baseline	4.85 ± 1.37	4.76 ± 1.73	0.23
Neutral (*Pottery*)	4.52 ± 2.03	5.00 ± 1.44	-1.07
Negative (*Curve*)	6.18 ± 1.59	6.55 ± 1.09	-1.05
**Resilience**	66.48 ± 14.08	69.17 ± 14.66	-0.74
**IU**	60.27 ± 16.72	58.90 ± 14.00	0.35
**Anxiety**	6.24 ± 3.25	8.31 ± 3.86	-2.29*
**Depression**	4.91 ± 3.89	4.31 ± 3.41	0.64
**Stress**	16.45 ± 6.13	17.10 ± 6.18	-0.41
**DERS**			
DERS-1	11.06 ± 3.59	13.24 ± 4.66	-2.08*
DERS-2	15.42 ± 4.15	14.93 ± 4.35	0.46
DERS-3	10.82 ± 4.05	10.17 ± 3.72	0.65
DERS-4	17.67 ± 4.61	13.21 ± 4.79	3.73***
DERS-5	15.70 ± 5.13	16.69 ± 5.71	-0.72
DERS-6	12.76 ± 3.73	10.86 ± 3.66	2.02*

Note: Resilience was measured by the Connor-Davidson Resilience Scale. Intolerance of Uncertainty (IU) was assessed by the Intolerance of Uncertainty Scale. Anxiety and depression were measured by the Hospital Anxiety and Depression Scale. Stress was measured with the Perceived Stress Scale. Emotion regulation problems were evaluated by the Difficulties in Emotion Regulation Scale (DERS). The DERS subscales from 1 to 6 represent the following difficulties in emotion regulation: 1- Nonacceptance of emotional responses; 2 - Difficulty engaging in goal-directed behavior; 3- Impulse control difficulties; 4 - Lack of emotional awareness; 5 - Limited access to emotion regulation strategies; 6 - Lack of emotional clarity. SD=standard deviation. *: p<0.05; **: p <0.01; ***: p<0.001.

Reference

Camacho, M. C., Karim, H. T., & Perlman, S. B. (2019). Neural architecture supporting active emotion processing in children: A multivariate approach. *NeuroImage*, *188*, 171–180. https://doi.org/10.1016/j.neuroimage.2018.12.013

Chen, P.-H. A., Jolly, E., Cheong, J. H., & Chang, L. J. (2020). Intersubject representational similarity analysis reveals individual variations in affective experience when watching erotic movies. *NeuroImage*, *216*, 116851. https://doi.org/10.1016/j.neuroimage.2020.116851

Girard, J. M. (2014). CARMA: Software for continuous affect rating and media annotation. *Journal of Open Research Software*, *2*(1), e5. https://doi.org/10.5334/jors.ar

Taylor, J. R., Williams, N., Cusack, R., Auer, T., Shafto, M. A., Dixon, M., Tyler, L. K., Cam-CAN, & Henson, R. N. (2017). The Cambridge Centre for Ageing and Neuroscience (Cam-CAN) data repository: Structural and functional MRI, MEG, and cognitive data from a cross-sectional adult lifespan sample. *NeuroImage*, *144*, 262–269. https://doi.org/10.1016/j.neuroimage.2015.09.018

van Baar, J. M., Halpern, D. J., & FeldmanHall, O. (2021). Intolerance of uncertainty modulates brain-to-brain synchrony during politically polarized perception. *Proceedings of the National Academy of Sciences*, *118*(20), e2022491118. https://doi.org/10.1073/pnas.2022491118
